# Porous PLLA microspheres dispersed in HA/collagen hydrogel as injectable facial fillers to enhance aesthetic effects

**DOI:** 10.1093/rb/rbaf049

**Published:** 2025-05-23

**Authors:** Miaoran Zhao, Shuhua Chang, Yunpeng Wang, Jun Cao, Yuji Pu, Bin He, Shengsheng Pan

**Affiliations:** National Engineering Research Center for Biomaterials, Collage of Biomedical Engineering, Sichuan University, Chengdu 610065, China; National Engineering Research Center for Biomaterials, Collage of Biomedical Engineering, Sichuan University, Chengdu 610065, China; National Engineering Research Center for Biomaterials, Collage of Biomedical Engineering, Sichuan University, Chengdu 610065, China; National Engineering Research Center for Biomaterials, Collage of Biomedical Engineering, Sichuan University, Chengdu 610065, China; National Engineering Research Center for Biomaterials, Collage of Biomedical Engineering, Sichuan University, Chengdu 610065, China; National Engineering Research Center for Biomaterials, Collage of Biomedical Engineering, Sichuan University, Chengdu 610065, China; Department of Plastic Surgery, First Affiliated Hospital of Wenzhou Medical University, Wenzhou 325000, China

**Keywords:** crosslinked HA/collagen hydrogel, porous PLLA microspheres, facial filler, collagen regeneration

## Abstract

Injectable facial fillers such as Sculptra^®^ stimulate collagen regeneration to fill wrinkles; however, the collagen regeneration is not satisfactory due to the slow emergence of filling effect. In this study, we designed a regenerative dermal filler to provide both immediate and long-lasting filling effects. A hydrogel matrix composed of crosslinked hyaluronic acid (HA) and collagen was engineered to encapsulate porous poly(L-lactide) (PLLA) microspheres and tranexamic acid (TXA). The hydrogel matrix was administered via intradermal injection to achieve wrinkle filling. TXA is released to exert skin-whitening effects, while the porous PLLA microspheres and their degradation product, lactic acid, continuously stimulate collagen regeneration over an extended period. Facial volume increased immediately following hydrogel injection. Large amounts of new Type I and Type III collagen are generated. The porous structure of PLLA microspheres facilitated the ‘penetrating growth’ of collagen fibers, which effectively filled facial depressions and smoothed wrinkles. Overall, the HA/collagen composite hydrogel filler exhibited excellent esthetic effects.

## Graphical abstract

**Figure:**
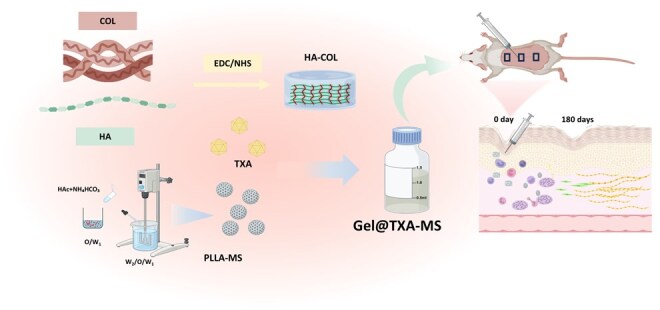

## Introduction

Aging including intrinsic aging and extrinsic aging occurs naturally over time. Intrinsic aging is a series of degenerative changes in biochemical molecules due to the progression of older age. Extrinsic aging is a biochemical process driven by external environmental, mechanical and lifestyle factors [1]. The aging process leads to the degeneration of physiological functions and biophysical properties of organs and tissues. The skin is a primary indicator of aging. The characteristics of facial skin aging include muscle and fat atrophy, skin laxity, age spots and sagging of the cheeks and eyelids [2, 3].

The strategies used for skin anti-aging are sun protection, radiofrequency, facial fillers, laser and so on [4]. Among them, facial fillers are mainly used to smooth out static wrinkles, improve facial contours and fill soft tissue defects on the face. Both absorbable and non-absorbable fillers are utilized [5]. The absorbable facial fillers include collagen, hyaluronic acid (HA), polylactic acid, etc. and the non-absorbable fillers are calcium hydroxyapatite, polymethyl methacrylate, silicone, etc. [6] Considering the occurrence of adverse reactions and the duration of the filling effects, the absorbable fillers are promising in improving static wrinkles on the skin. The essence of static wrinkle is due to the rapid loss of Type I collagen. The injection of facial collagen filler is a direct way to replenish collagen. The collagen-based skin fillers appeared in the 1970s, however, the *in vivo* proteases degraded the injected collagen rapidly within 3–6 months, thus, other products such as crosslinked HA hydrogels were developed to replace injectable collagens.

HA-based fillers were introduced in the 1990s, the excellent biocompatibility would not cause allergic reactions when implanted in patients [7, 8]. The high water-binding capacity of HA fillers made them excellent volumizing agents for soft tissues. Thus, the HA-based fillers are one of the widely used anti-aging materials [9] and hyaluronidase can be used to degrade HA for reversible regulation when embolism occurs [10]. Both collagen and HA could restore the volume and elasticity of soft tissues through injection. The HA fillers are degraded by endogenous enzymes in the skin and do not match the long-lasting filling expectation as wrinkle fillers. Therefore, it is necessary to improve traditional wrinkle fillers to maximize their lifespan in the dermal tissues [11] with good biocompatibility, non-toxicity and excellent mechanical properties [12].

PLLA particles such as Sculptra^®^ from France, AestheFill^®^ from South Korea and Derma Veil^®^ from the United States are new types of biodegradable facial fillers [13]. In 2021, the first ‘PLLA Facial Filler (Rejuvenation Needle)’ was officially approved by the National Medical Productions Administration (NMPA) of China. Different from collagen and HA fillers, PLLA can be fabricated into microspheres and used to load active ingredients for the preparation of facial fillers, so as to improve facial aesthetic effects [14–16].

In the early stage of PLLA post-injection, proteins absorbed on the microspheres and macrophages gather around them to generate slight inflammation. It stimulates the aggregation and proliferation of fibroblasts to secrete collagen to supplement the lost collagen in aging. As a result, the skin thickness gradually increases to smoothing wrinkles and filling depressions [13, 17–19]. However, the regeneration of collagen could not exhibit prompt filling effects, to achieve the rapid filling effects and long-lasting filling effects at the same time, we intend to combine the hydrogel of crosslinked collagen and HA and PLLA microspheres together to fabricate a new regenerative facial filler that addresses the disadvantages of Sculptra^®^, such as severe inflammatory reactions [20] due to its irregular particle structure and slow stimulating collagen regeneration [21].

**Figure 1.**
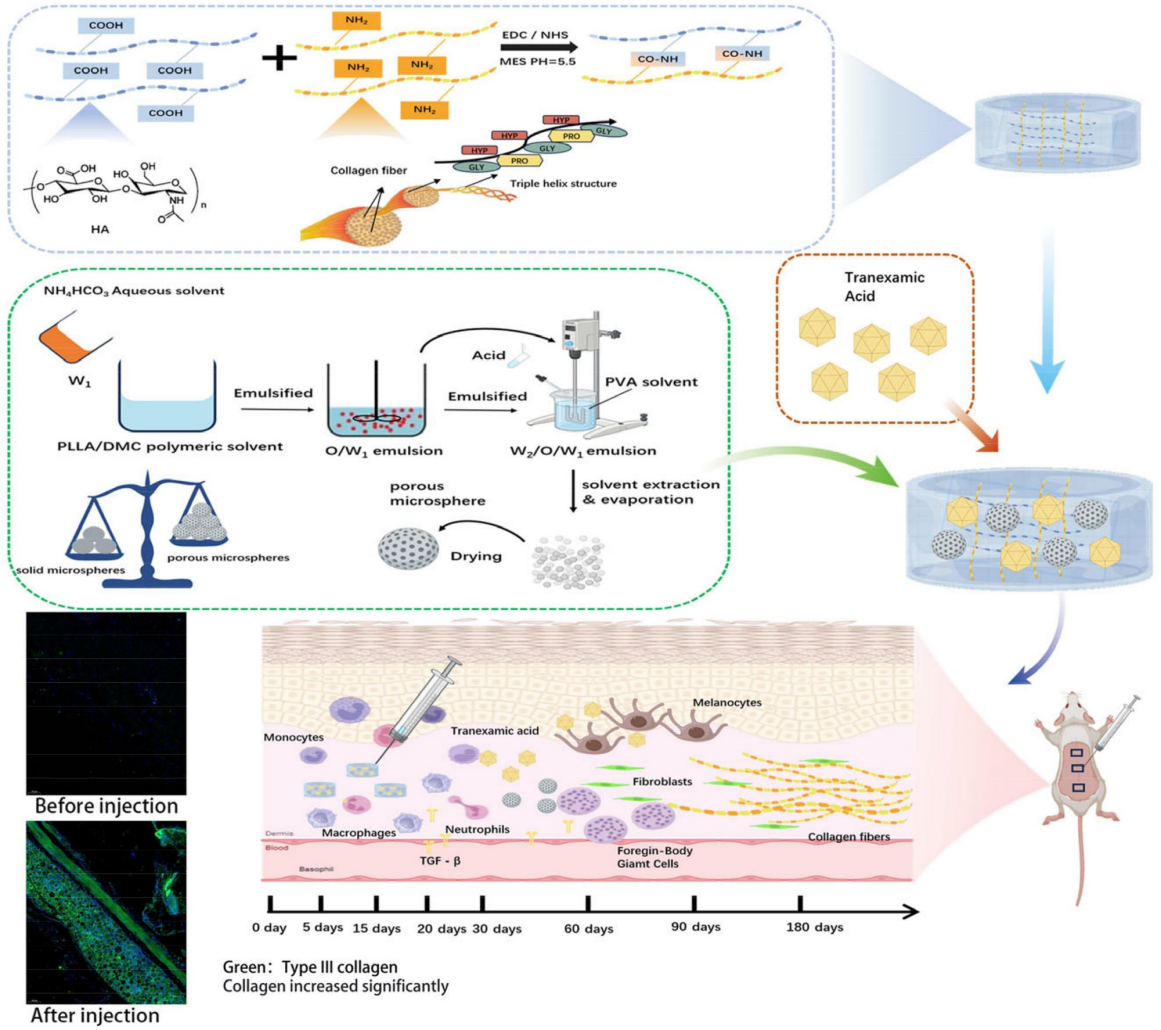
Schematic illustration of porous PLLA microspheres dispersed in HA/collagen hydrogel as the injectable facial filler.

In this article, 3D network hydrogels of HA and collagen were prepared using 1-ethyl-3-(3-dimethylaminopropyl)carbodiimide and N-hydroxy succinimide as crosslinking agents. The collagen was introduced to improve mechanical strength and cell affinity in the HA/collagen hydrogels [22]. The tranexamic acid (TXA) was loaded in the hydrogel to offer a whitening effect. The porous PLLA microspheres dispersed in the hydrogels enabled better cellular attachment and proliferation to accelerate the collagen regeneration [23, 24]. As shown in Figure 1, the facial fillers of HA/collagen hydrogels together with porous PLLA microspheres achieved both the prompt and long-lasting filling effects.

## Experimental section

### Materials

1-Ethyl-3-(3-dimethylaminopropyl)carbodiimide (EDC, 95%), Type I bovine tendon collagen (S12007) were purchased from Yuanye Biotechnology Co., Ltd (Shanghai, China). N-Hydroxy succinimide (NHS, 98%) was purchased from Adamas Reagent Co., Ltd (Shanghai, China). Poly(L-lactide) (MW = 100 000 Da) was synthesized in our laboratory. HA (MW = 20 000 Da) and polyvinyl alcohol (PVA) with a viscosity range of 25.0–31.0 mPa s were purchased from Aladdin Biochemical Technology Co., Ltd (Shanghai, China). Tranexamic acid (98%, TXA) was purchased from Macklin Biochemical Co., Ltd (Shanghai, China). Six-week-old Sprague-Dawley (SD) rats were purchased from Gem Pharmatech Co., Ltd. RAW264.7 macrophages were provided by the National Certified Cell Culture Collection Center. Mouse fibroblasts (L929) were provided by the American Type Culture Collection (ATCC).

### Characterizations

The morphologies of freeze-dried hydrogels, microspheres and composite fillers were observed under a scanning electron microscope (SEM, Model S4800, Hitachi, Japan). The samples were fixed by conductive adhesive, gold sputtering treatment was carried out on a sputtering coater and the samples were analysed at 20 kV. The particle size and size distribution of the microspheres were measured on a Microtrac particle size analyzer (McCeek Instruments Co., Ltd, Model S3500-SI). Statistical analysis was carried out with ImageJ software. The rheological properties of the hydrogels were tested on an Anton Paar MCR 302 rheometer (Anton Paar Trading Co., Ltd) using a PP25 plate (with a diameter of 25 mm and a gap of 1 mm). The measurement temperature was set at 37°C. Five hundred microliters of samples were taken and added to the exact center of the plate. The viscosity changes of hydrogels under shear stresses ranging from 1 to 100 were investigated. The rheology of polymers under an oscillation frequency of 1 Hz and a shear strain (γ) of 1% was measured at 37°C, the shear strain was increased from 0.1% to 100% while keeping the angular frequency at 10 rad/s. The strain sweep test on the hydrogels was conducted with the strain cycle scans of 1% and 1000% at 37°C at 10 rad/s. High-performance liquid chromatography (HPLC) was performed using equipment from Shimadzu Instruments (Suzhou) Co., Ltd, SPD-16. The chromatographic conditions were set as follows: a Diamonsil C18 chromatographic column (250 mm × 4.6 mm, 5 μm) was used with the mobile phase of pH = 2.5 phosphate buffer—methanol (60:40, v/v), the flow rate was set at 0.8 mL/min, the detection wavelength was set at 220 nm, the injection volume was set at 20 μl and the column temperature was set at 30°C. UV spectra were measured on an ultraviolet spectrophotometer (Model 302, Anton Paar, Austria). Optical graphs were taken on an optical microscope (Model DMI 4000, Leica Instrument Co., Ltd, Germany). Flow cytometry was carried out using a flow cytometer (BD Medical Devices Co., Ltd, USA). Fourier transform infrared (FT-IR) spectra were recorded using a Thermo Nicolet (NEXUS 670) via a potassium bromide approach.

### Preparation of composite facial fillers

#### Preparation of hydrogel

About 0.976 g of 2-morpholinoethanesulfonic acid (MES) was accurately weighed, 50 mL of ultrapure water was added and the mixture was stirred until it was completely dissolved. The pH is adjusted to 5.5 by dropwise adding 1 M NaOH. This buffer solution was used to respectively prepare a HA solution with a concentration of 10 mg/mL and a volume of 20 mL, as well as a collagen solution with a concentration of 20 mg/mL and a volume of 20 mL. An environment at 4°C was used to place the above two solutions, and they were stirred evenly. About 1.52 g EDC and 0.228 g of NHS (nEDC:nNHS = 4:1) were added to the solution, and the solution was stirred slowly for 12 h to form a gel. The gel was dialyzed in ultrapure water (MWCO = 5000 Da) to have the crosslinking agents removed. The ultrapure water was replaced every 4 h, and the dialysis lasted for 24 h. The dialyzed hydrogel was stored at 4°C for later use.

#### Preparation of microspheres

The emulsification—solvent evaporation method was adopted to fabricate porous PLLA microspheres. About 0.8 g of PLLA was dissolved in 16 mL of dichloromethane to form an oil phase (O) with a concentration of 5% (w/v). Four milliliters of a 10% (w/v) ammonium bicarbonate (NH_4_HCO_3_) aqueous solution (W_1_) were added to the oil phase, and ultrasonic treatment was carried out to form a water-in-oil system (O/W_1_). The mixture and 200 μl of glacial acetic acid (HAc) were added to 200 mL of a 2% PVA aqueous solution (W_2_) under a stirring speed of 1200 rpm to form a water-in-oil-in-water system (W_2_/O/W_1_). After 30 min, the stirring speed was adjusted to 800 rpm. The dichloromethane was evaporated to obtain porous microspheres. The microspheres were washed five times each with ultrapure water and ethanol. The products were freeze-dried for later use.

#### The dispersion of microspheres in hydrogel

About 0.8 g of the lyophilized microspheres and 1.2 g of TXA are weighed, and they are added to 40 mL of the crosslinked hydrogel. The concentration of microspheres was set at 20 mg/mL, and that of TXA was set at 30 mg/mL. The mixture was slowly stirred until homogeneous. After being sterilized by irradiation, the injectable filler of porous PLLA microspheres in HA/collagen hydrogel (Gel@TXA-MS) was obtained.

### Water content

The hydrogels with different crosslinking degrees were taken to investigate the absorbed water content. The surface moisture absorbed was cleaned with filter paper. The weight was recorded as *M*_0_. The samples were put into liquid nitrogen and freeze-dried for 24 h. The freeze-dried weight was recorded as *M_h_*. Under the same conditions, the water content test was conducted three times. The water content of hydrogels was calculated using the following formula:


$$\mathrm{Water}\;\mathrm{content}\;(\%)=\frac{M_{0}-M_{h}}{M_{0}}\times 100\%$$

### Injectability and stability

The Gel@TXA-MS filler was aspirated using a 26 G needle, and the Sculptra^®^ filler was aspirated using a 25 G needle. The universal testing machine (Zhiqu Precision Instruments Co., Ltd, ZQ 990LB) was used to test the extrusion force of the products at an extrusion speed of 30 mm/min to evaluate the injectability of the filler and the situation of needle blockage. The Gel@TXA-MS filler and Sculptra^®^ were left standing for 12 h, and the deposition of microspheres was observed to evaluate the stability of the system.

### Release profile of TXA

#### Standard curve of TXA

Ten milligrams of TXA were weighed and dissolved in phosphate buffered saline (PBS) so that a stock solution with a concentration of 1 mg/mL was prepared. Then the stock solution was diluted to have test solutions with a concentration range of 0.5–5 mg/mL. The tests were carried out on a HPLC. The peak area *y* of the characteristic peak in the chromatogram was recorded, and the linear regression of *y* against the concentration *c* was conducted to obtain the standard curve. See Figure S2 in the Supporting Information (SI) for details.

#### In vitro drug release

Five milliliters of drug-loaded hydrogel were put in a dialysis bag with a molecular weight cut-off of 3000 D. The bag was soaked in 20 mL of PBS buffer solution and placed in a shaking incubator at 37°C. At predetermined time points, 1 mL of the external solution was taken out for HPLC detection. Three parallel samples were set up at each time point. The longest time point was set as 3 days. One milliliter of fresh PBS was added to replenish the media. The drug release curve was obtained according to the chromatogram detection at different time points.

### *In vitro* and *in vivo* degradation of microspheres

#### *In vitro* degradation

Ten milligrams of microspheres were added to tubes with 2 mL of PBS buffer solution containing 0.2 mg/mL elastase. The tubes were placed in a water bath shaker at 37°C. At regular intervals, one tube with microspheres was taken out, centrifuged at 4000 rpm for 5 min to have the supernatant removed, the microspheres were washed with ultrapure water three times and freeze-dried. The morphology of the degraded microspheres was observed using a scanning electron microscope. The fresh PBS was replaced once a week and the tubes were replaced once every two weeks.

#### *In vivo* degradation

About 0.2 mL of the filler was subcutaneously injected into SD rats. At predetermined time points, the rats were sacrificed, the skin was taken for embedding and sectioning, and stained with hematoxylin and eosin (H&E) to have the degradation of microspheres *in vivo* observed.

### Cytotoxicity

#### MTT assay

Hundred milligrams of solid MTT were dissolved in 20 mL of PBS buffer solution. The solution was sterilized via a filter with a pore size of 0.22 μm and stored at −20°C away from light. The MTT solution was diluted 10 times with serum-free medium. L929 cells in the logarithmic growth phase were seeded into 96-well plates at a density of 5 × 10³–1 × 10^4^ cells per well. After the cells have been permitted to attach to the bottom of the plate overnight, the blank culture medium was replaced with the culture medium containing the materials. The blank plate as utilized as the control. Hundred microliters of the material-containing culture medium or the blank culture medium were added to each well. The sample groups of HA/collagen hydrogel (Gel), microspheres (MS), hydrogel with TXA (Gel@TXA) and hydrogel with TXA and MS (Gel@TXA-MS) were placed in 96-well plates. The concentrations of PLLA microspheres were set at 50, 100, 200, 300 and 400 μg/mL, respectively. For the groups with hydrogels, the concentrations of the freeze-dried hydrogel powders were set at 50, 100, 200, 300 and 400 μg/mL, respectively. Five parallel replicates were prepared for each concentration. After being incubated for 48 and 72 h, then the medium was aspirated, the cells were washed with PBS twice, 100 μl of serum-free medium containing 5 μg/mL MTT staining solution was added to the wells and incubated at 37°C for 4 h. After the medium was aspirated, 100 μl of DMSO was added to each well to dissolve the produced formazan. The plates were shaken and allowed to react for 2 min. A microplate reader was used to detect the ultraviolet absorbance at 490 nm. The final experimental results were reflected by the mean value and standard deviation, and the cell survival rate was calculated according to the following formula:


$$\mathrm{Cell}\;\mathrm{viability}\;(\%)=\frac{A_{x}-A_{b}}{A_{c}-A_{b}}\times 100\%$$

where *A_x_* was the absorbance of the DMSO solution in the material group, *A_b_* was the absorbance of the DMSO solution in the blank group (the group without cells) and *A_c_* was the absorbance of the DMSO solution in the control group.

#### Calcein/PI cell viability and cytotoxicity detection assay

L929 cells in the logarithmic growth phase were seeded into 12-well plates at a density of 1 × 10^5^–1 × 10^6^ cells per well. After the cells were allowed to adhere to the plates overnight, the culture medium was replaced with the extract of the culture medium containing the materials. The control group, Gel group, MS group, Gel@TXA group and Gel@TXA-MS group were established. Each well was filled with 1 mL of culture medium, and three replicate wells were set for each group. In the Gel group and the Gel@TXA group, the lyophilized hydrogel powder was set at a concentration of 400 μg/mL. In the MS group and the Gel@TXA-MS group, the lyophilized microspheres were set at a concentration of 400 μg/mL. After being incubated for 48 h and 72 h, respectively, the culture medium was aspirated, and the cells were washed twice with PBS. Subsequently, 5 μl of Calcein-AM solution (2 mM) and 15 μl of PI solution (1.5 mM) were taken and added to 5 mL of 1×Assay Buffer, and the mixture was thoroughly blended. Calcein/PI detection working solution (500 μL) was added, and the samples were incubated at 37°C in the dark for 30 min. The staining situation of the cells was observed by means of an inverted fluorescence microscope.

### Antioxidant properties

During the logarithmic growth phase, RAW264.7 cells were seeded into 6-well plates at a density of approximately 3 × 10^5^–5 × 10^5^ cells per well. After the cells were adhered to the plate, 2 mL of culture medium containing 100 μM hydrogen peroxide (which was required to be diluted in a gradient) was added to the sample group and the hydrogen peroxide group. After incubated for 6 h, the culture medium was aspirated to stimulate the production of reactive oxygen species (ROS), and the residual hydrogen peroxide was washed with phosphate buffered saline (PBS). One milliliter of the Gel@TXA-MS filler was soaked in 4 mL of complete medium for 3 days to ensure that the concentration of TXA was 30 mg/mL after it was completely released, and then it was filtered through a 0.22-μm filter. Subsequently, 2 mL of the extract was added to the cells in the sample group. Two milliliters of fresh complete medium were added to the hydrogen peroxide group, and no treatment was administered to the control group. The cells in each group were co-cultured for 24 h. The culture medium was aspirated, and the cells were treated with 10 μM 2,7-dichlorofluorescein diacetate (DCFH-DA) for 30 min and then washed twice with PBS. The cells were collected and analysed by flow cytometry. The percentage of positive events in the fluorescein isothiocyanate (FITC) channel was recorded, and the data analysis was carried out using Flow Jo software.

#### DPPH scavenging assay

Two milligrams of DPPH were weighed and dissolved in 50 mL of absolute ethanol. The composite facial filler was diluted with ultrapure water and stood for 48 h to ensure the complete release of TXA. The concentration range was 5–40 mg/mL when TXA was completely released. The solution was filtered to remove the microspheres and avoid affecting the subsequent ultraviolet absorption results. Two milliliters of samples at each concentration were prepared for later use. The operation was carried out in a 96-well plate away from light. Hundred microliters of sample solution and 100 μl of DPPH ethanol solution were added to the sample group; 100 μl of sample solution and 100 μl of absolute ethanol were added to the blank group; 100 μl of deionized water and 100 μl of DPPH ethanol solution were added to the control group. Three replicate wells were set up for each group. After the corresponding samples were added, the mixtures were incubated at room temperature away from light for 30 min, and the absorbance at 517 nm was detected with a microplate reader. The DPPH scavenging efficiency of each concentration was calculated according to the following formula and a line chart was drawn


$$\mathrm{Scavenging}\;\mathrm{rate}\;（\%）=1-\frac{A_{\mathrm{sample}}-A_{\mathrm{blank}}}{A_{\mathrm{control}}}\times 100\%$$

#### The ability of the composite facial filler to inhibit melanin

##### The inhibition of cell proliferation

The composite fillers were soaked in complete medium for 3 days and filtered with a 0.22 μm sterile filter, and the maximum concentration was made to 80 mg/mL. L929 cells and B16 cells in the logarithmic growth phase were inoculated into 96-well plates at the density of 5 × 10³–1 × 10^4^ cells per well. After the cells were adhered to the wall overnight, the medium was replaced with the medium containing materials, and 100 μl of the medium was added to each well. In addition, for each 96-well plate, a negative control group without adding materials and a blank control group without cells and materials were set up. The concentrations of TXA in the material group were 80, 60, 40, 20, 10 and 5 mg/mL, respectively. The subsequent steps were the same as those in the MTT assay.

##### Influence on the morphology of B16 cells

The composite fillers were soaked in complete medium for 3 days and filtered with a 0.22-μm sterile filter, the concentrations of TXA were 0, 20, 30 and 40 mg/mL, respectively. B16 cells in a good growth state were digested into a cell suspension and inoculated into 12-well plates at the density of 1 × 10^5^–1 × 10^6^ cells per well. After the cells were adhered to the plate, 1 mL of the prepared extract containing different concentrations of TXA was added to each well, and the cells were then cultured continuously for 48 h. The medium was aspirated, and washed with PBS twice. Finally, 500 μl of PBS was added to maintain the integrity of the cell morphology. The morphology of B16 cells was observed with an inverted electron microscope.

##### Detection of melanin content

The composite fillers were soaked in complete medium for 3 days and filtered with a 0.22-μm sterile filter. B16 cells in the logarithmic growth phase were digested with trypsin and then inoculated into 12-well plates at the density of 1 × 10^5^–1 × 10^6^ cells per well, the cells were continuously cultured after 1 mL of culture medium was added to each well. When the cells were adhered to the wall, 1 mL of the extract with a TXA concentration of 30 mg/mL was added to the experimental group, 1 mL of the extract with the ascorbic acid concentration of 30 mg/mL was added to the positive control group, and a blank control group was set up. The cells were treated for 72 h. Three parallel samples were set for each group. After the materials were taken effect, an equal amount of B16 cells was collected from each well, washed twice with PBS and centrifuged at a speed of 1000 rpm for 5 min. Three hundred microliters of ultrapure water were added to the cell pellet to resuspend the cells, and 1 mL of an ethanol/ether mixed solution (*v*:*v* = 1:1) was added and stood for 15 min. The mixture was centrifuged with a speed of 3000 rpm for 5 min, and the supernatant was discarded. One milliliter of 1 mol/l sodium hydroxide solution containing 10% dimethyl sulfoxide (DMSO) was slowly added to the cells. After fully shaken, the cells were incubated at 80°C for 30 min to lyse the cells and dissolve the intracellular melanin. The supernatant was transferred to a 96-well plate, and the absorbance (OD) was measured at a wavelength of 470 nm using a microplate reader. Three replicate wells were set for each group, and the experiment was repeated three times. The melanin content and the melanin inhibition rate were calculated according to the following formulas, and a line chart was drawn


$$\mathrm{Melanin}\;\mathrm{content}=\frac{{\mathrm{OD}}_{\mathrm{sample}}\times 10^{6}}{\mathrm{numble}\;\mathrm{of}\;\mathrm{cells}}$$

$$\mathrm{Melanin}\;\mathrm{inhibition}\;\mathrm{rate}（\%）=\frac{1-{\mathrm{OD}}_{\mathrm{sample}}}{{\mathrm{OD}}_{\mathrm{control}}}\times 100\%$$

### *In vivo* filling effect

For the *in vivo* experiments, Group A was the injectable porous polylactic acid microsphere composite hydrogel filler (Gel@TXA-MS), Group B was the Sculptra^®^ filler and Group C was the control group injected with normal saline (Control). In these experiments, female SD rats weighing about 200 g were used as experimental subjects. A total of 10 time points (5, 10, 15, 20, 30, 60, 90, 120, 150, 180 days) were set, and three parallel rats were in each time point. The rats were raised for 3 days to adapt the environment. The hair on the backs of the rats was shaved and disinfected. About 0.2 mL of the samples was subcutaneously injected into three different parts on the backs of the rats using a 26 G syringe. After injection, the symptoms such as erythema and edema on the back skin were regularly observed. At each predetermined time point, the rats were sacrificed for subsequent histological observation. All animal studies were carried out in accordance with the ‘Animal Management Regulations of the Ministry of Health of the People's Republic of China’ (No. 55 in 2001) and institutional guidelines, and approved by the Animal Care and Use Committee of Sichuan University.

The full-thickness skin tissues of the rats were fixed with 4% paraformaldehyde tissue fixative for 48 h, embedded in paraffin and sectioned. H&E was used for subsequent histological analysis. Nucleic acid structures in the cell nucleus were stained purple or blue by hematoxylin, and the proteins in the basic structures were stained pink or red by eosin, which was conducive to the intuitive analysis of the distribution of subcutaneous materials in rats, the degree of material degradation and the degree of immune response; and analyzed using Image J software. Three sample values of the full-thickness skin thickness at the site of the injected material and the blank site in the H&E staining images were measured by ImageJ software. These values were used to quantify the increase in skin thickness, and a bar chart was drawn using Origin. After the tissues were fixed in the same way, Masson's trichrome staining (Masson) was carried out to investigate the generation and distribution of collagen as collagen fibers or proteins were stained blue and myofibroblasts or proteins were stained purple. After the tissues were fixed, fluorescence staining treatments for CD68, IL-6, Type I collagen and Type III collagen were carried out. Observations were made through a fluorescence microscope, and semi-quantitative analysis of the results was conducted using ImageJ software. The proportion of the fluorescently positive area was statistically analyzed, and the statistical results of three repetitions were carried out in different regions of the same section. CD68 is a marker of immune response. The specific binding on the surfaces of antigens and antibodies is helpful for the localization and identification of macrophages and assists in analyzing the degree of immune response. As a key pro-inflammatory cytokine, the dynamic changes of IL-6 can reflect the intensity of the inflammatory response and tissue compatibility. Through the fluorescence staining treatments of Type I collagen and Type III collagen, the types and distributions of collagen generated in different periods were determined.

### Statistical analysis

The experimental data in this study are all based on at least three parallel experiments to ensure their reliability and representativeness. The data are presented in the form of ‘Mean ± SD’, which intuitively reflects the central tendency and dispersion degree of the data. One-way analysis of variance (ANOVA) was used to evaluate the significant differences among the data of different groups to determine whether the sample means were from the same population. In the statistical results, if *P < 0.05, **P < 0.01, then ***P < 0.001 indicates a statistically significant difference, while n.s. means no significant difference. These criteria are widely applied in academic research and effectively verify the scientific nature of the experimental results.

## Results and discussion

### Characterizations of hydrogels

**Figure 2.**
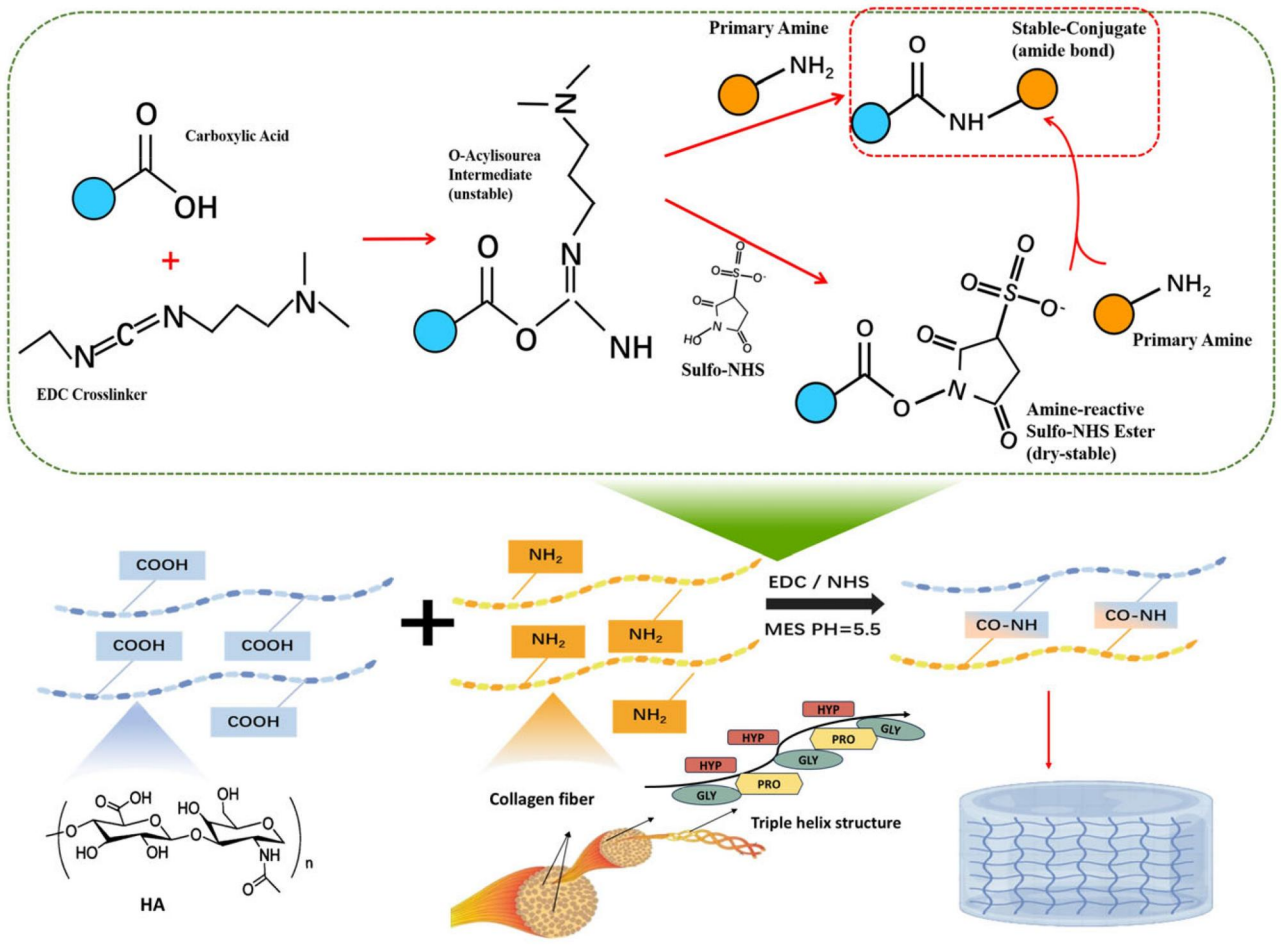
The synthesis of HA/collagen hydrogel.

Collagen and HA were crosslinked to form hydrogel using EDC/NHS as catalysts. The synthetic route is shown in Figure 2 [25–28]. The hydrogel underwent dialysis for purification [29].

**Figure 3.**
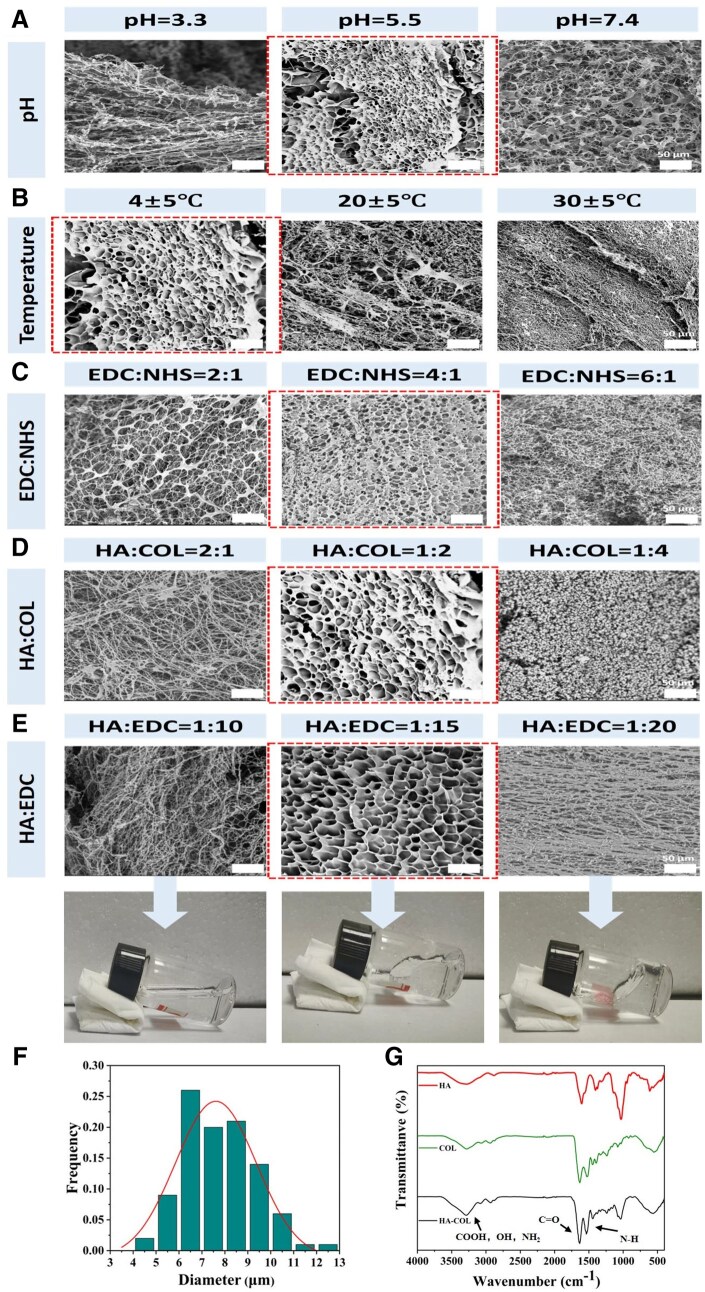
Characterization of crosslinked HA/collagen hydrogels. (**A**)–(**E**) SEM images of hydrogels synthesized under different reaction conditions. The more ideal results are outlined by a dashed box. (**F**) Pore size distribution maps of the target hydrogels. (**G**) FT-IR spectra of crosslinked HA/collagen hydrogels.

The factors to affect the hydrogel formation were investigated. The morphologies of the freeze-dried hydrogels were observed by SEM (Figure 3). As shown in Figure 3A, when the pH value was 5.5, a more uniform network structure was formed compared with other conditions. As EDC exhibited the highest activity at pH of 4–6, the MES buffer solution with pH 5.5 was used as the medium for crosslinking reaction [28]. Three temperatures of 4, 20 and 30°C were selected for considering the reaction. At 4°C, at least 12 h were necessary for the formation of hydrogel [30–32]. As shown in Figure 3B, disordered fibrous structures were observed at 20 and 30°C, which implied that the crosslinking was not occurred, thus, 4°C was used as the reaction temperature, highlighted by a red square. In Figure 3C, the molar ratio between EDC and NHS was investigated. When the ratio of *n*(EDC):*n*(NHS) was 4:1, a more uniform network structure was formed. We chose the ratio of *n*(EDC):*n*(NHS) as 4:1 for the next step of the experiment [31]. As shown in Figure 3D, when the ratio of *n*(HA):*n*(COL) was 1:2, a stable network structure was presented. While the ratio of *n*(HA):*n*(EDC) was 1:15, the hydrogel was suitable for injection (Figure 3E). The more ideal states are all circled by red squares. Thus, the factors were set as pH 5.5, 4°C, EDC:NHS = 4:1, HA:COL = 1:2 and HA:EDC was 1:15. The SEM images under this condition are shown in the red square frame in Figure 3E. The pore diameter was approximately 8 μm (Figure 3F) and the porosity was 80%.

The freeze-dried samples of COL, HA and HA-COL were weighed, respectively, and subjected to FT-IR detection. The results are shown in Figure 3G. With the addition of EDC and NHS, an increase in the amide bond at 1650 cm^−1^ in the hydrogel was clearly observed, accompanied with the decrease in the carboxyl and amino groups at 3100–3500 cm^−1^. It implied the formation of hydrogel as the porous structure shown in SEM images [30, 33].

**Figure 4.**
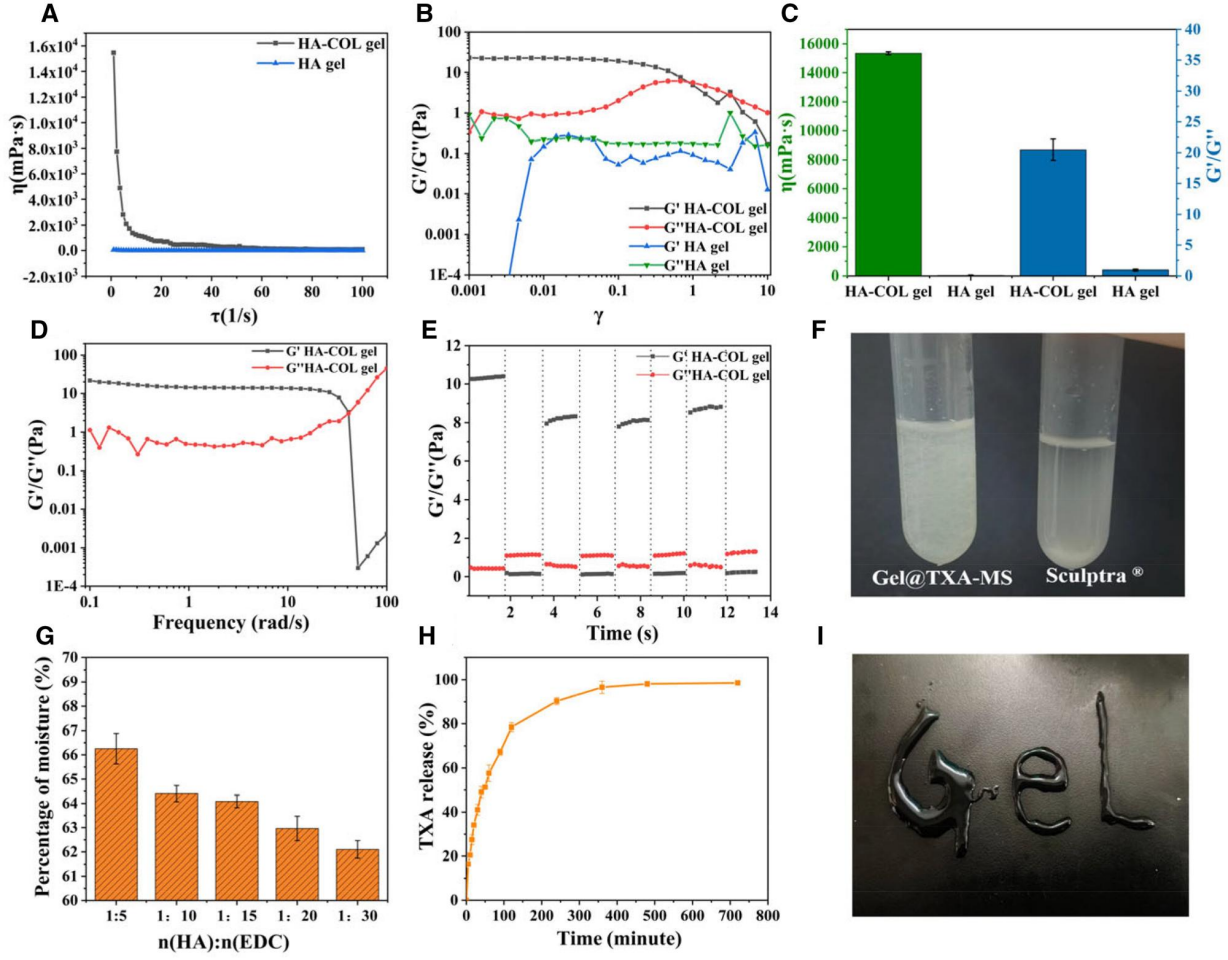
Characterizations of crosslinked HA/collagen hydrogels. (A) Viscosity changes of hydrogels under shear stress ranging from 1 to 100 Pa. (B) Changes in the modulus of hydrogels under shear strain ranging from 0.1% to 100%. (C) Comprehensive comparison of the mechanical properties of two types of hydrogels. (D) Variation in the modulus of hydrogels across different angular frequencies. (E) Investigation of the self-healing properties of hydrogels under cyclic strain scanning at 1% and 100%. (F) Investigation of the stability of hydrogels. (G) Statistical analysis of the water content of crosslinked hydrogels with different proportions. (H) The release curve of TXA detected by HPLC. (I) Injectability of crosslinked HA/collagen hydrogel.

To further evaluate the rheological properties and injectability of the hydrogel, we used a rheometer to analyze the dynamic rheological properties of the hydrogel. The temperature was at 37°C, and the viscosity changes of the hydrogel were examined under a shear stress ranging from 1 to 100 Pa (Figure 4A). Compared with the HA gel material, the viscosity of the HA-COL crosslinked hydrogel was significantly increased. It provided good stability for the composites as facial fillers with the addition of PLLA microspheres, once the fillers were injected under the epidermis, it was beneficial for the adhesion of fibroblasts with collagen regenerative to enable more precise filling of depressed areas [34]. Shear strain was increased from 0.1% to 100% with a constant angular frequency of 10 rad/s. A strain sweep test was conducted on the hydrogel (Figure 4B). The elastic modulus (*G*′) of the crosslinked hydrogel was improved to a certain extent. The increase in the elastic modulus meant that the crosslinked hydrogel possessed more excellent elasticity, stronger anti-deformation ability and stable mechanical properties. Both collagen and crosslinking stabilized the hydrogel [35, 36]. In Figure 4C, a comprehensive comparison of the two materials also revealed that the crosslinked hydrogel facial filler had a better volumizing effect and facial lifting ability compared to the single HA gel filler. As shown in Figure 4D, when the angular frequency increased, the crosslinked injectable hydrogel maintained its good solid-state characteristics, *G*′ was greater than *G*″. In addition, the hydrogel also possessed excellent injectable properties (Figure 4I). In addition, the fibrous structure of the hydrogel, the heterogeneity of the gel and the particle size of the microspheres may lead to blockage when passing through a thin needle. The injection force of the filler can be accurately measured by a universal testing machine, as shown in Supplementary Figure S1. Since the Sculptra^®^ filler is severely blocked in a 26 G syringe and cannot be pushed out, the average extrusion force of the Sculptra^®^ filler in a 25 G syringe is measured to be 0.84 N. The average extrusion force of the Gel@TXA-MS filler in a 25 G syringe is 0.46 N, and the average extrusion force in a 26 G syringe is 0.87 N. The injection force of the filler is a key factor, and it can be normally used clinically when the force is below 10 N. These results indicate that the Gel@TXA-MS filler can smoothly pass through a 26 G needle without blockage, showing good injectability. Figure 4E illustrates the self-healing property of the hydrogel to potentially achieve ideal filling effect [26, 33, 37, 38]. In Figure 4F, the HA-COL crosslinked hydrogel with PLLA microspheres addition maintained uniform hydrogel state even stewing for 12 h, however, the PLLA microspheres in the aqueous solution of Sculptra^®^ filler were deposited at the bottom with 12 h stewing. Due to the confinement effect provided by the 3D network structure of the hydrogel, the microspheres are ensured to be uniformly dispersed without obvious aggregation. The specific surface area of the porous PLLA microspheres is larger than that of the solid microspheres, resulting in a larger interaction area with the hydrogel matrix and a stronger confinement effect. In addition, the porous PLLA microspheres have a relatively low density and lighter mass, and are less affected by gravity, so they are not prone to sedimentation in the mixed system, thereby maintaining uniform dispersion.

The water content of different hydrogels could reach above 60% (Figure 4G). With the increase of the crosslinking degree, the pore diameter of the hydrogel decreased and the water-holding capacity also declined [39]. The higher crosslinking degree compacted the space in the hydrogel and resulted in lower water content. To a facial filler product, the hydrogel with relatively higher water content and stable structure is preferable.

To investigate the controlled release of TXA from the hydrogel, HPLC was used for detection. Based on the standard curve of TXA (Supplementary Figure S2), the drug release curve is plotted as shown in Figure 4H [40, 41]. At 120 and 480 min, the cumulative release amounts of TXA reached 80% and 99%, respectively. TXA was distributed in the network structure of the hydrogel and the load active ingredients would be beneficial to facial fillers [42].

### Porous PLLA microspheres

**Figure 5.**
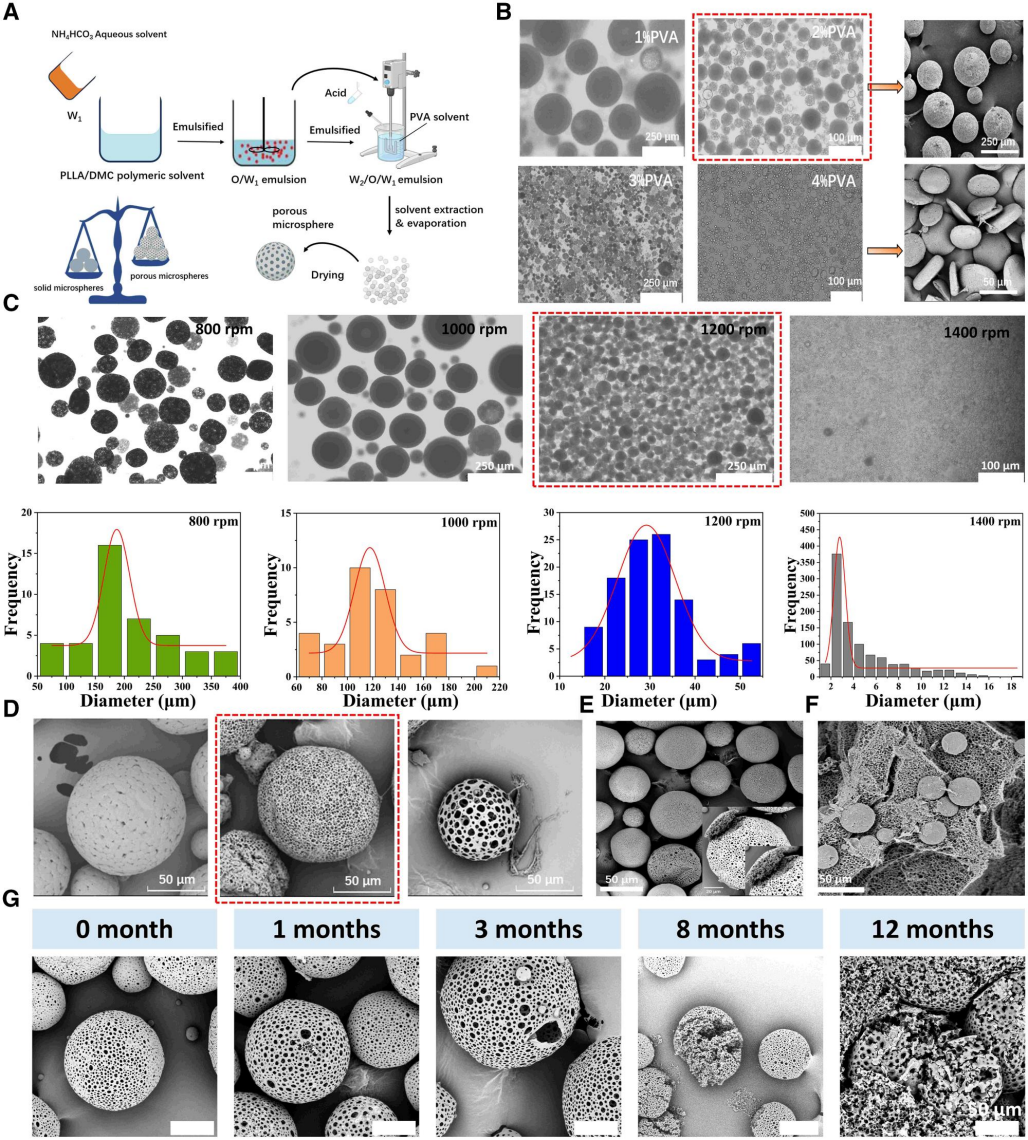
Characterization and degradation behavior of porous microspheres. (**A**) Flow diagram showing the preparation of porous microspheres. (**B**) Electron microscope images and SEM morphological images of microspheres prepared with PVA concentration of 1%, 2%, 3% and 4%. (**C**) Electron microscope images and particle size distribution diagrams of microspheres at rotational speeds of 800, 1000, 1200 and 1400 rpm. (**D**) SEM images of microspheres under three pore-forming methods. (**E**, **F**) SEM images of the final porous microspheres and Gel@TXA-MS filler. (**G**) SEM images of the degradation behavior of microspheres *in vitro* within one year.

The emulsion solvent evaporation method is adopted to prepare porous PLLA microspheres (Figure 5A) [43–45]. The microspheres with different particle sizes could be obtained by adjusting the stirring rate (Figure 5C). The average particle size of the microspheres could be decreased from about 200 μm to around 2 μm [46]. When the concentration of PVA increased, the particle size of microspheres decreased. Once the concentration of PVA was too high, spherical microspheres could not be obtained by this method due to excessive resistance to sphere formation, and disc-shaped microspheres were formed instead (Figure 5B) [47]. Three pore-forming methods were explored. As shown in Figure 5D, from left to right in sequence: only 10% (w/v) NH_4_HCO_3_, 10% NH_4_HCO_3_ and 0.01% (w/v) HAc, 10% NH_4_HCO_3_ and 0.02% (w/v) HAc. The pore size of the microspheres increased accordingly. When HAc was not added, the intensity of the thermal decomposition of NH_4_HCO_3_ was relatively low, while the chemical reaction between NH_4_HCO_3_ and HAc generated more CO_2_ bubbles, thus a large amount of CO_2_ was generated to form honeycomb-like porous microspheres [43, 45]. In conclusion, under the conditions of a rotation speed of 1200 rpm and a PVA concentration of 2%, by adding 10% NH_4_HCO_3_ and an equal volume of HAc, porous honeycomb-like microspheres with a size in the range of 20–70 μm were prepared [19]. The microspheres with the morphology shown in Figure 5E were selected. Furthermore, the morphology image of the obtained composite facial filler (Gel@TXA-MS) is shown in Figure 5F. When the particle size of the microspheres was too small, they were easily recognized and phagocytosed by immune cells. If the particle size was too large, it would cause needle blockage, which was not convenient for clinical operations. In addition, the pore size of the porous microspheres was approximately in the range of 2–5 μm. The honeycomb-like structure was beneficial to the inward growth of cells, and the newly regenerated collagen could also penetrate through the interior of the microspheres, which was more helpful for targeted filling of facial depressions and achieving ‘controlled regeneration’ [48]. The porous honeycomb-like microspheres could provide larger volume for the attachment of fibroblasts to generate better filling effects [49, 50].

The degradation of PLLA microspheres is shown in Figure 5G. In 1 month, the microspheres maintained their intact morphology, and no obvious degradation was observed. In 3 months, the microspheres exhibited degradation and damage as some larger pores appeared. In 8 months, the microspheres were further degraded, and the spherical structure was damaged. In 12 months, no spherical structure was observed, the porous sheet-like structure was dispersed. It implied that the facial filler degraded slowly and could stimulate the regeneration of collagen for a long period of time [17, 19, 51].

### Biocompatibility of the composite facial fillers

**Figure 6.**
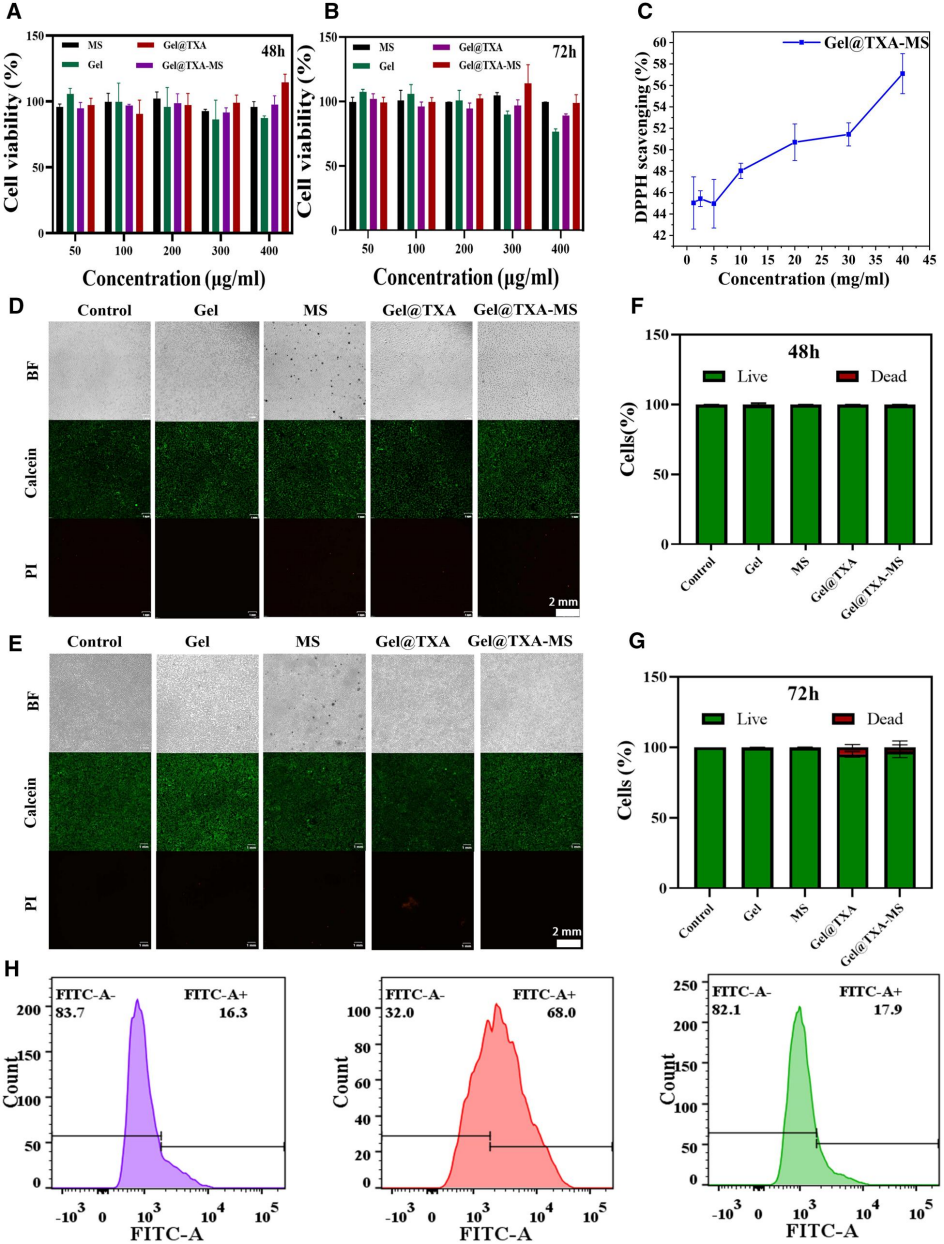
Cytotoxicity and antioxidant properties of Gel@TXA-MS filler. (**A**, **B**) Statistical analysis of cytotoxicity detected by MTT assay at 48 and 72 h. (**C**) ROS scavenging efficiency of the composite facial fillers investigated by the DPPH method. (**D–G**) Live-dead cell staining images and corresponding quantitative analysis after 48 and 72 h of treatment. (**H**) Detection of the intracellular ROS scavenging efficiency by flow cytometry.

The extract of the facial filler was incubated with L929 cells for 48 and 72 h to investigate the biocompatibility. As shown in Figure 6A and B, when the concentrations of fillers were 400 μg/mL, both cell survival rates were above 85%, indicating excellent biocompatibility. The results of live/dead cell viability staining are shown in Figure 6D and E, only a few individual cells showed red fluorescence. Figure 6F and G are the statistical charts of live/dead cells at 48 and 72 h. Most of the cells were in a good viability state.

### Antioxidant properties of the composite facial fillers

1,1-Diphenyl-2-picrylhydrazyl (DPPH) is a stable free radical with a nitrogen center and strong absorption at 517 nm. Therefore, the DPPH method was used to investigate the ROS scavenging characteristics of the composite facial filler [52]. The results are shown in Figure 6C. As the concentration of TXA increased, the scavenging effect on DPPH increased successively. When the concentration of TXA in the composite filler was higher than 2%, the effect was more prominent, and the free radical scavenging efficiency could reach above 50%. TXA exerts a reductive effect by transferring electrons and protons to the single electron on the nitrogen atom of DPPH to generate DPPH_2_ [53]. One of the important reasons for human aging is the generation of free radicals. The free radicals accelerate the aging of cells in the body. Therefore, the composite facial filler with TXA would delay the cell aging to achieve better esthetic effect [54].

Inspired by the results of the DPPH experiment, we further investigated the *in vitro* ROS scavenging ability. The extracts of the composite fillers were co-cultured with macrophages. The sample, positive control and blank control were set up. After the cells were treated with DCFH-DA, the analysis was carried out by flow cytometry and Flow Jo software. The results are shown in Figure 6H. Compared with the positive control group, the fluorescence intensity of the sample group was significantly reduced, and the ROS-positive cell rate in the positive control group decreased from 68% to 17.9%. This indicated that the sample possessed excellent ROS scavenging ability [55].

When the expression of some antioxidant-related genes is downregulated and the antioxidant defense is overwhelmed by ROS, ROS will attack biological macromolecules such as lipids, proteins and DNA within the cell. This will lead to lipid peroxidation, altering the fluidity and permeability of the cell membrane. Once the protein is oxidized, its structure and function will be affected. Oxidative damage to DNA may cause gene mutations, chromosomal aberrations and so on. Oxidative stress will occur in the cellular environment, leading to tissue decline and an accelerated aging process. Through the comprehensive analysis of the DPPH experiment and the ROS scavenging experiment, it is known that the addition of TXA endows the facial filler with excellent antioxidant properties, delaying cell aging from the root. In addition, by scavenging ROS and maintaining the vitality of fibroblasts, it is more conducive for PLLA microspheres to promote collagen regeneration. This broadens the anti-aging dimension of the facial filler. The synergistic anti-aging effect of ‘passive filling’ and ‘active repair’ is of great significance in the field of facial fillers.

### The ability of the composite facial filler to inhibit melanin

**Figure 7.**
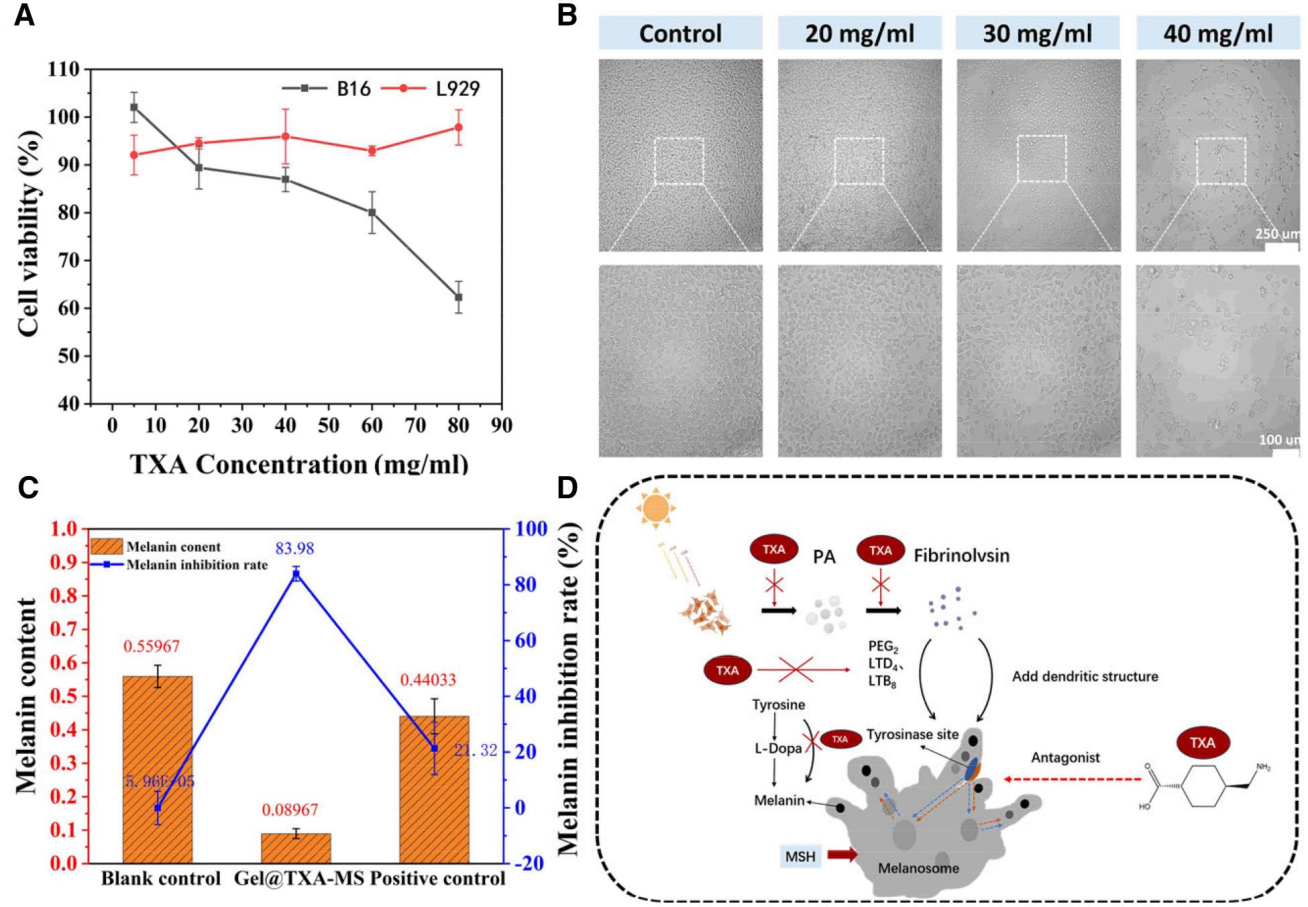
Evaluation of the effect of inhibiting melanin of composite facial fillers. (A) Statistical charts of the viability of B16 and L929 cells treated with TXA at different concentrations. (B) Morphology observation of B16 cells treated with TXA at different concentrations. (C) Quantitative analysis of the intracellular melanin content and the melanin inhibition rate in B16 cells after treatment with the materials. (D) Schematic illustration of the melanin inhibition mechanism of TXA.

The melanin synthesis function of B16 cells is very similar to that in human skin. Therefore, we selected B16 cells to evaluate the effect of inhibiting melanin. After the L929 and B16 cells were co-cultured with the extracts of the composite filler at different concentrations for 72 h, the MTT assay was used to monitor the growth status of the two types of cells to evaluate the effect of inhibiting melanin. As shown in Figure 7A, the survival rates of both types of cells were above 85% without extract. When the concentration of the material increased, the L929 cells still maintained a relatively high survival rate, while the growth of B16 cells was significantly inhibited. When the concentration of TXA reached 80 mg/mL, the survival rate of B16 cells was only about 60%. There was a statistically significant difference in the growth status of the two types of cells (*P *< 0.001). In the same way, the extract of the composite filler was co-cultured with B16 cells, and the morphology of B16 cells was observed using an inverted fluorescence microscope. The mature normal melanocytes were epithelia cells with a dendritic structure. The dendritic structure plays a crucial role in melanin production, transport and deposition. Therefore, examination of the cell morphology is of great significance for evaluating the effect of inhibiting melanin. In the normal B16 cells in Figure 7B, most cells had a symmetrical dendritic structure, and more irregular protrusions appeared with further cell culture. However, compared with normal cells and the cells with TXA addition, the proliferation of B16 cells was significantly inhibited and the cell viability also decreased. The dendritic structure was shortened or even disappeared, and a round cell structure was formed. The addition of TXA inhibited the function of melanin secretion and hindered the formation of the dendritic structure of melanocytes, thereby inhibiting the establishment of a melanin unit with surrounding keratinocytes and exhibiting a certain impact on the melanin transport process [56]. TXA is a protease inhibitor, and it also has an inhibitory effect on the activity of tyrosinase, which is essential for melanin production. The principle of its action is shown in Figure 7D [57]. The effect of inhibiting melanin has precious value in today's esthetic trend [58].

In Figure 7C, the melanin content in the group of the composite facial filler containing TXA (Gel@TXA-MS) decreased, and the melanin inhibition rate reached 83.98%, four times higher than that of the ascorbic acid group (positive control). The effect of inhibiting melanin was obviously better. On the one hand, TXA has a similar structure to that of tyrosine and directly competes with tyrosine to reduce the catalytic effect of tyrosinase on tyrosine and thus reducing melanin production, it also reduces plasmin activity, inhibits keratinocytes from releasing prostaglandin E_2_(PGE_2_) to reduce melanin production; it activates the autophagy system and reduces melanin content, moreover, TXA is a protease inhibitor and inhibits the transport of melanin to keratinocytes [59–61].

### *In vivo* evaluation of the composite facial filler

#### Cytotoxicity

To evaluate the histocompatibility of the filler, a subcutaneous injection model in rats was used for the study. At two time points, the 15th day and the 30th day after the subcutaneous injection, tissue samples were, respectively, obtained and processed by the H&E staining method. By observing the stained tissue samples, the results are shown in Supplementary Figure S6. It can be clearly seen that the cell composition in each tissue exhibited a significant and regular arrangement pattern. Meanwhile, in these tissue samples, there are no signs of abnormal conditions such as necrosis, bleeding or inflammatory exudates. Throughout the entire test cycle, the full-thickness skin of the injection site of the experimental rats was taken at different time points. As shown in Supplementary Figure S7, there were no other abnormal conditions such as erythema and edema on the surface and inside. This indicates that the Gel@TXA-MS and Sculptra^®^ fillers have good biocompatibility. Based on the above observation results, it can be concluded that this filler is not toxic to tissues.

#### Immediate filling effect

**Figure 8.**
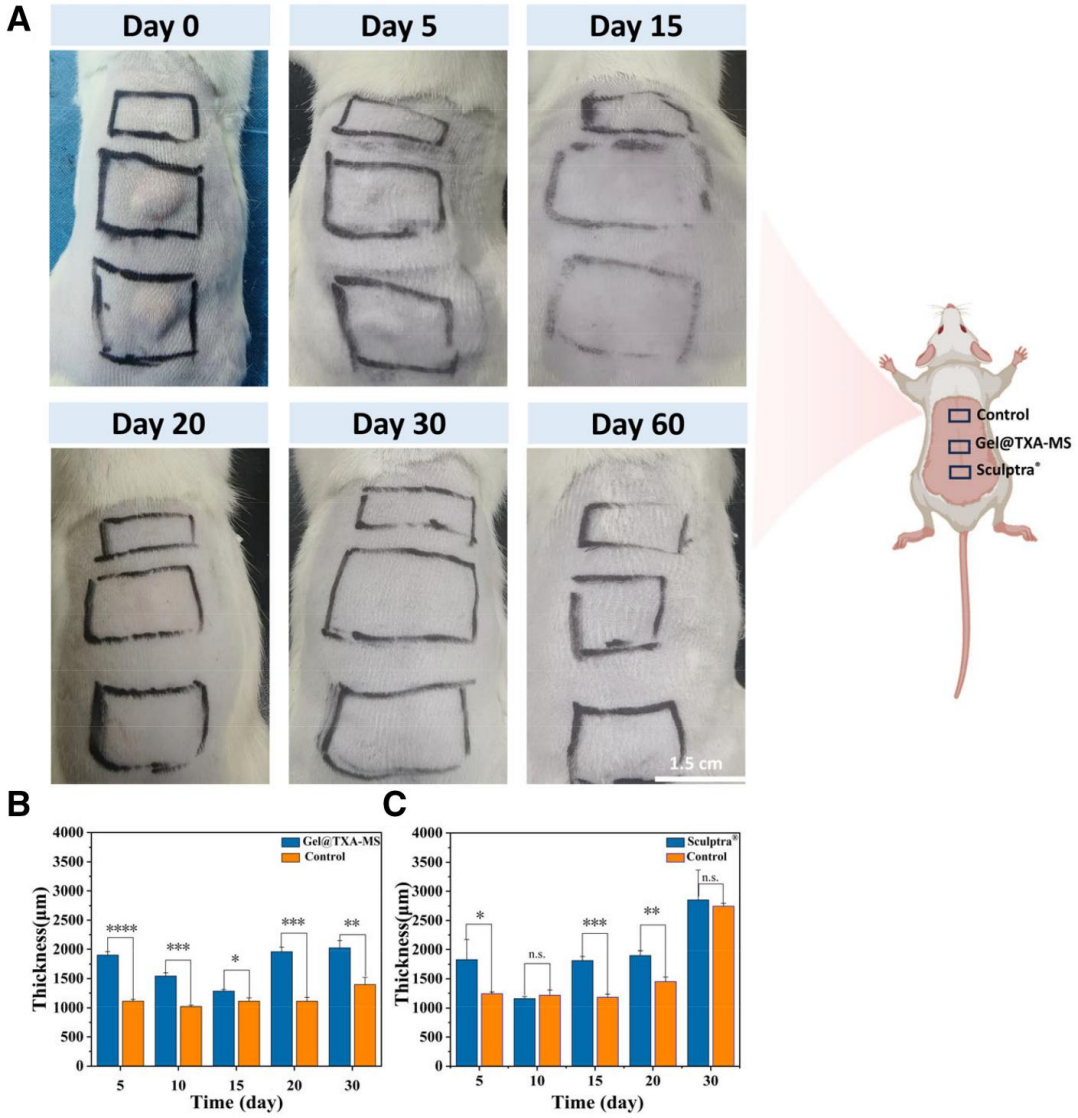
(**A**) The diagram of immediate filling effect in animal experiments. (**B**, **C**) Quantitative analysis of immediate filling effect. *P < 0.05, **P < 0.01 and ***P < 0.001.

No obvious difference was observed in the back skin of rats injected with Gel@TXA-MS and Sculptra^®^ fillers within 0–15 days (Figure 8A). After 15 days, the visible protrusion gradually disappeared. From 15 to 30 days, a certain protrusion could still be felt at the injection site subcutaneously, and it gradually disappeared after 30 days (Figure 8A and Supplementary Figure S3). As shown in Figure 8B and C, the back skin of rats with Gel@TXA-MS filler injection showed a stable increase in thickness, which was better than that of Sculptra^®^ filler [62]. The mechanical stability and water content of the Gel@TXA-MS filler were better than those of carboxymethyl cellulose (CMC) in Sculptra^®^ filler, thus, the immediate filling effect of Gel@TXA-MS is better than that of Sculptra^®^ filler.

#### Inflammatory response and *in vivo* degradation

**Figure 9.**
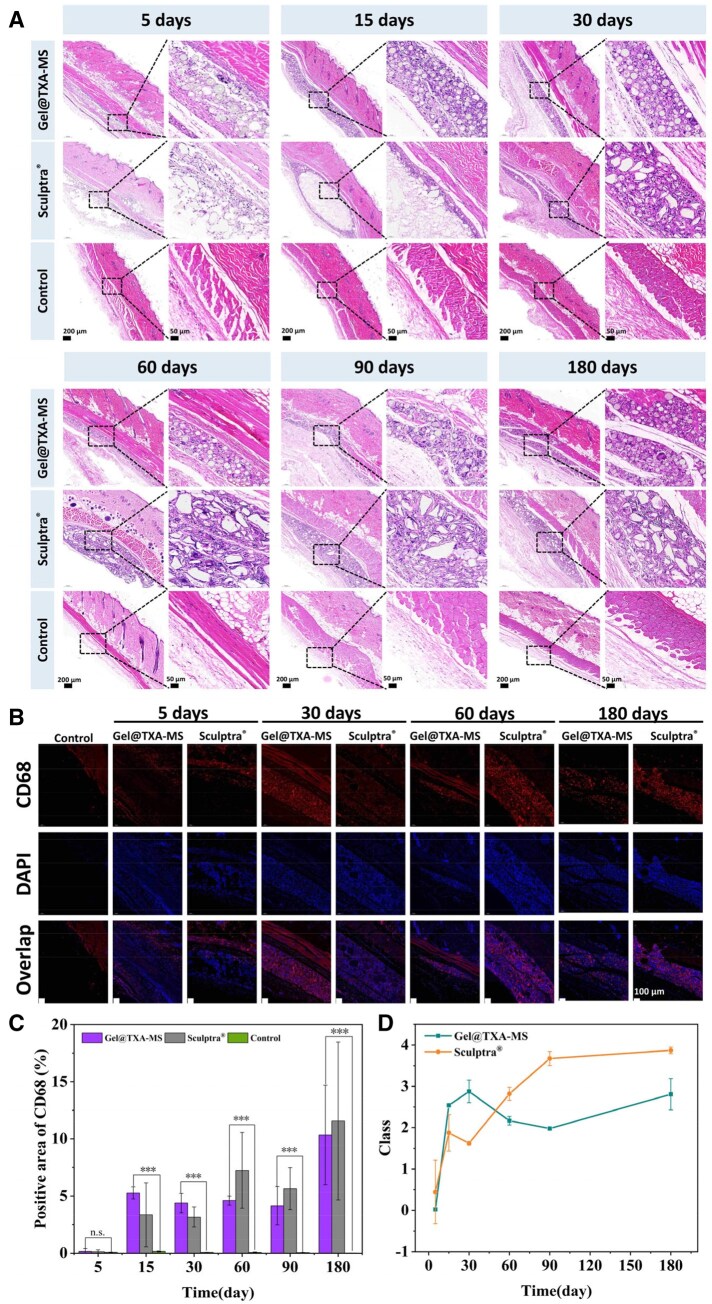
Inflammatory response. (**A**) H&E-stained sections collected from day 5 to day180. (**B**) Fluorescence images of CD68 in tissue sections. (**C**) Quantitative analysis of CD68 fluorescence staining. (**D**) Comparative analysis of inflammatory responses by two filling materials. *P < 0.05, **P < 0.01 and ***P < 0.001.

From the H&E sections of Gel@TXA-MS filler injection on the fifth day (as shown in Figure 9A), there were many porous microspheres with a diameter of about 50 μm in the subcutaneous tissue, which were evenly distributed without obvious aggregation. With the occurrence of the foreign body reaction (FBR), some inflammatory cells were observed gathering around the foreign substances, and some neutrophils with trilobed or bilobed nuclei were seen. In addition, combined with the results of CD68 immunofluorescence (Figure 9B), the red fluorescence around the microspheres was weak and the number of macrophages was still small, the fluorescence mainly appeared around the entire materials [63]. In the H&E section of Sculptra^®^ filler on the fifth day, uneven distribution, agglomeration phenomena and large blank areas could be clearly seen, and the tissue edema occurred. On the 15th day, many immune cells migrated. Around Gel@TXA-MS filler, macrophages invaded the pores and migrated toward the inside of the microspheres. The same result could be seen in the CD68 staining image (Supplementary Figure S8), where there was a large amount of red fluorescence around the microspheres, and the macrophages were wrapped around the microspheres and even migrated into the interior of the material. The fibrous encapsulation area formed by Sculptra^®^ filler was relatively large, with less PLLA materials and immune cells inside, and immune cells have infiltrated around it. From the CD68 immunofluorescence staining image (Supplementary Figure S8), it could also be seen that there were almost no cells inside the material, and only some red fluorescence appeared around the material. On the 30th day, more immune cells such as macrophages, neutrophils and monocytes infiltrated around the microspheres. As macrophages were unable to phagocytize the microspheres, many foreign body giant cells (FBGCs) were formed by fuzing with the microspheres under the action of IL-4 or/and IL-13. With the microsphere’s degradation, the pore sizes gradually increased and the PLLA microspheres began to break, allowing immune cells to enter the interior of the microspheres. In the CD68 fluorescence images, the red fluorescence was significantly enhanced and distributed more densely, indicating the further increase of immune cells. Through the location analysis of the cells in combination with the DAPI images, it was found that most of the cells were wrapped around the microspheres, and weak red fluorescence appeared inside the microspheres, suggesting that macrophages had invaded the interior of the microspheres and the inflammatory response had reached a more intense stage. In contrast, to Sculptra^®^ filler, the site of the inflammatory response was only concentrated at the edges of the material. In the CD68 images, the red fluorescence was only presented around the material, mainly neutrophils while few FBGCs were observed. After subcutaneous implantation in rats for 60 and 90 days, the inflammatory reaction around Gel@TXA-MS filler was significantly weaker than that around Sculptra^®^ filler (Figure 9A). Many inflammatory cells gathered around Sculptra^®^ filler. Due to the implantation of foreign substances, a series of moderate chronic inflammatory reactions occurred, multinucleated giant cells appeared and accumulated to produce collagen. From the immunofluorescence images, the degree of the inflammatory reaction of Gel@TXA-MS filler was equivalent to the previous level, while that of Sculptra^®^ filler was aggravated, large number of immune cells were wrapped around the material. The H&E staining results at other time points are shown in Supplementary Figure S4. After implanted for 180 days, both materials have spread to some extent with the movement of the rats, Gel@TXA-MS filler was more evenly distributed. The degradation degree of the microspheres of Gel@TXA-MS filler increased. The degradation of PLLA in Sculptra^®^ filler was not obvious and no immune cells were seen entering the interior of the microspheres. In the CD68 immunofluorescence staining image (Figure 9B), the red fluorescence in Gel@TXA-MS filler was relatively evenly distributed around and inside the microspheres, while in Sculptra^®^ filler, the red fluorescence was densely concentrated around the material, and the fluorescence intensity was stronger than that of Gel@TXA-MS filler, indicating a more intense inflammatory reaction. The quantitative statistical chart of CD68 fluorescence is shown in Figure 9C. The appropriate size and regular shape of Gel@TXA-MS filler avoided an overly concentrated inflammatory response, and the overall degree of inflammatory response was lower.

Immunofluorescent staining of IL-6 was carried out to further evaluate the inflammatory response of the filler. As shown in Supplementary Figure S9. Macrophages are one of the main source cells of IL-6. When PLLA microspheres are implanted into the body, they will stimulate macrophages to activate the intracellular signaling pathways, prompting them to secrete IL-6. IL-6 can not only promote the activation and proliferation of macrophages and regulate the inflammatory response, but also promote the proliferation of fibroblasts, enabling them to synthesize more collagen. In the Gel@TXA-MS group, on the 15th, 30th and 60th days after the implantation of PLLA microspheres, the levels of IL-6 increased significantly, but the inflammatory level remained stable throughout. Subsequently, the level of IL-6 gradually declined on the 90th day and returned to a lower value on the 180th day. This changing pattern in which the level of IL-6 first increases and then decreases while the inflammation remains stable fully indicates that this facial filler can induce a moderate and controllable inflammatory response, reflecting good biocompatibility and will not lead to severe persistent immune responses. In contrast, the expression level of IL-6 in the Sculptra^®^ group is higher, and the results of the fluorescent staining are more concentrated. On the 180th day, IL-6 still has a high expression. Therefore, the expression level of IL-6 in the Gel@TXA-MS filler is lower. A low level of IL-6 brings many benefits: it reduces the incidence of inflammatory responses such as redness and swelling, decreases the risk of excessive fibrosis, and at the same time ensures a more natural filling effect, avoiding abnormal tissue hyperplasia or deformation caused by excessive inflammation.

Referring to the criteria for the severity of subclinical FBR, the degree of FBR of the two fillers was evaluated. The biodegradation process of PLLA was divided into two main stages. The first stage was closely related to the subclinical FBR: after the implantation, the body absorbed proteins and triggered the FBR. In the initial stage, neutrophils among immune cells first infiltrated into the peripheral tissues of the polymer, and then many monocytes poured in, triggering an inflammatory reaction, and promoting the up regulation of cytokine expression. Monocytes then differentiated into macrophages. Since the foreign body was too large for macrophages to phagocytose completely, they fused with each other to form multinucleated FBGCs. During this process, angiogenesis was also induced, and fibroblasts were activated to synthesize collagen, which finally wrapped the polymer and led to fibrosis. The formed capsule was mainly composed of Type I and Type III collagens, and the thickness was positively correlated with the ratio of the surface area to the volume of the polymer. The second stage: the intensity of the immune effect gradually weakened, and the inflammatory reaction and immune cells gradually decreased. FBGCs played a role in the degradation process of the polymer and continue to exist throughout the life cycle of the implantation [17, 64].

Duranti *et al.* established criteria for evaluating the severity of subclinical FBRs, which cover different levels of severity: (0) no visible reaction; (1) sparse inflammatory cells; (2) 1 or 2 giant cells; (3) fibrous tissue with inflammatory cells, lymphocytes, and giant cells; (4) granulomas with capsules [65, 66]. The two materials were analyzed quantitatively according to the scoring criteria, combined with H&E and CD68 fluorescence staining, and the results are shown in the Figure 9D. Gel@TXA-MS filler reached level 3 at 1 month, containing a large number of inflammatory cells, and the degree of the inflammatory reaction reached its maximum. As time progressed, the degree weakened but remained between level 2 and level 3. Sculptra^®^ filler had a slower increase in the inflammatory reaction in the early stage, and the distribution of inflammatory cells was uneven. Many inflammatory cells gathered at the sharp corners of the PLLA material. It reached level 3 at 2 months, then continued to increase, reached level 3.6 at 3 months, and reached level 4 at 6 months, with a significant increase in CD68 fluorescence intensity. In comparison, the degree of the inflammatory reaction of material B was more intense. However, the current classification method regarded all these levels as mild, which met the inflammatory criteria for facial filling. From a histological perspective, the multi-level classification of the severity of FBR is of great value and has reference value in clinical practice.

#### Collagen regeneration effect

**Figure 10.**
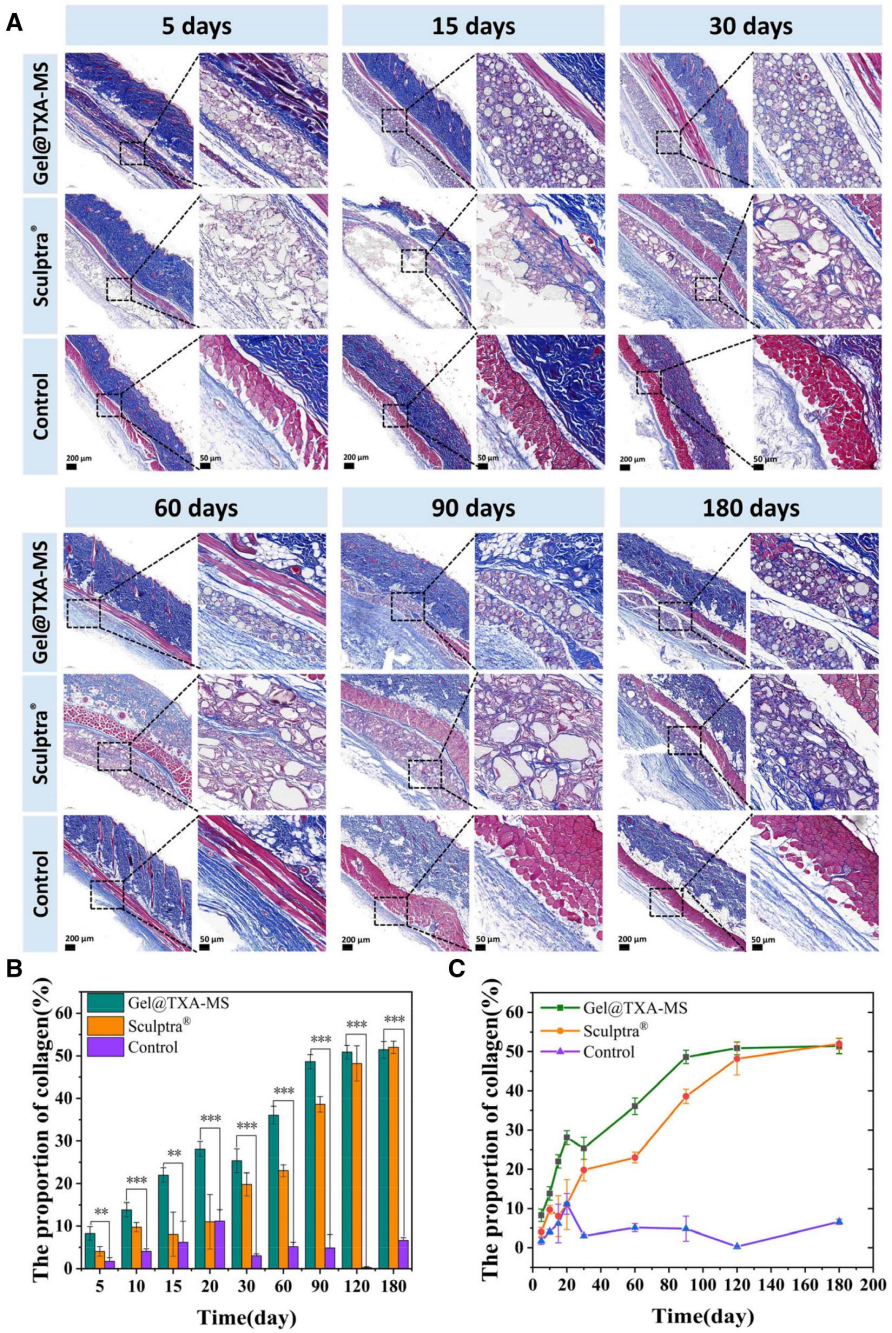
Collagen regeneration effect. (**A**) Masson-stained sections collected from day 5 to day180. (**B**) Quantitative analysis of collagen regeneration. (**C**) Trend charts of collagen regeneration. *P < 0.05, **P < 0.01 and ***P < 0.001.

The analysis was conducted in combination with the Masson staining images (Figure 10A and Supplementary Figure S5). On the fifth day, the collagen fibers in both fillers were relatively thin and appeared blue, while the muscle fibers were relatively thick and had a fixed orientation. At this stage, which was in the early period after injection, no obvious newly formed collagen fiber bundles were observed around either Gel@TXA-MS filler or Sculptra^®^ filler. On the 15th day, there were relatively thin blue fiber bundles interspersed around the microspheres in Gel@TXA-MS filler and distributed very evenly and orderly. However, as the encapsulation of Sculptra^®^ filler was too large, resulting in relatively large internal voids and no arrangement of fiber bundles. On the 30th day, the amount of light blue fibrous substances around Gel@TXA-MS filler increased and wrapped around the microspheres due to the migration of fibroblasts around the microspheres and production of collagen under the stimulation of biological factors secreted by macrophages. In contrast, the effect of Sculptra^®^ filler on stimulating collagen production was far inferior to that of Gel@TXA-MS filler. The distribution was uneven, and only some blue fiber bundles could be seen around the material. On the 90th day, more blue fibrous substances were generated. Compared with the data on the 30th day, it could be seen that the color of the fibrous substances became darker, and they were thicker. The quantitative statistics of collagen production are shown in Figure 10B. The quantitative analysis was performed using ImageJ software to calculate the area proportion of the blue fiber bundles in the Masson staining result images. The differences in the proportion of collagen fiber bundles between the two facial fillers and the control group were comparatively analyzed, so as to quantitatively analyze the collagen regeneration effects of the two fillers. At 3 months, the collagen production of Gel@TXA-MS filler and Sculptra^®^ filler could reach 48.6% and 38.5%, respectively. Overall, in terms of the early collagen production situation, the level of Gel@TXA-MS filler was stably higher than that of Sculptra^®^ filler. On the 180th day, blue collagen fiber bundles were distributed around both Gel@TXA-MS filler and Sculptra^®^ filler. Quantitative analysis showed that the levels of the two fillers were equivalent, reaching 51.4% and 52.0%, respectively. As the degradation degree of PLLA microspheres increases, the immune response gradually intensifies. More inflammatory cells accumulate at the injection site, stimulating the proliferation of fibroblasts, and the amount of collagen deposition also gradually increases. The amount of collagen deposition matches the degradation time of PLLA microspheres. According to Figure 10C, the Gel@TXA-MS filler has a generally more excellent collagen regeneration effect, which was attributed to the porous and regular spherical structure. It stimulated the production of more collagen fibers and enabled them to grow inward and penetrate. It is conducive to the localized generation of collagen and more advantageous for achieving the ideal facial filling effect [67].

#### Collagen types

**Figure 11.**
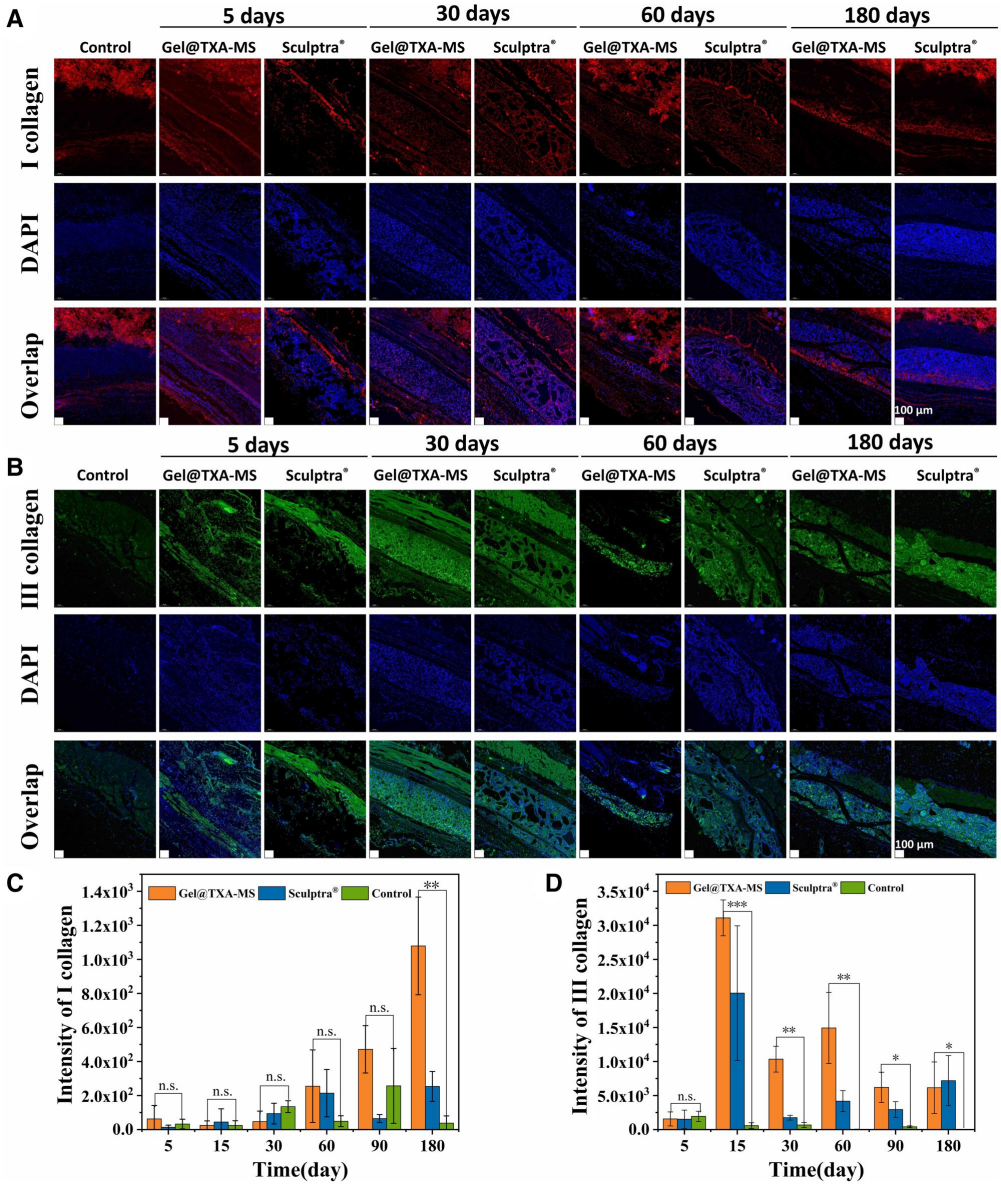
(**A**, **C**) Fluorescence images of Type I collagen staining and quantitative analysis at 180 days. (**B**, **D**) Fluorescence images of Type III collagen staining and quantitative analysis at 180 days. *P < 0.05, **P < 0.01 and ***P < 0.001.

To further characterize the types of collagens produced subcutaneously in rats, we performed two kinds of immunofluorescence staining treatments, namely Type I and Type III collagens, on the full-thickness skin sections of the rats in the experimental groups. The results are shown in Figure 11A and B and Supplementary Figures S10 and S11. Type I collagen fibers are relatively thick, providing the toughness of the skin but lacking elasticity, and mainly play a role in supporting the contour of the skin. Type III collagen fibers make the skin elastic and tender but lack toughness [68]. Through the fluorescence staining of Type I collagen and Type III collagen, the types and conditions of collagen production were further determined, which provided certain reference significance for the subsequent targeted partial filling in clinical use.

On the fifth day, the fluorescence intensities of both types of collagens were relatively weak. In the fluorescence staining image of Gel@TXA-MS filler, from the fifth to the 30th day, with the aggregation of fibroblasts and degradation of PLLA microspheres to stimulate fibroblasts for collagen production, the fluorescence intensity of Type III collagen around the microspheres increased rapidly, which was 53.3 times higher than that of the blank control group (Figure 11D). It was mainly distributed around the microspheres, and there was less Type I collagen. As time progressed to the 180th day, the appearance time of Type III collagen fluorescence was earlier than that of Type I collagen, and it always maintained a relatively strong fluorescence intensity. Green fluorescence could also be seen inside the microspheres. The fluorescence intensity of Type I collagen was relatively weak in the early stage. It was 1 month time that red fluorescence could be seen around the microspheres. At 2 months, both the intensity and quantity of the red fluorescence increased, showing a dotted distribution around the microspheres. At 3 months, red fluorescence could be seen in a sheet-like distribution around the microspheres. At 6 months, Type I collagen increased significantly, and the red fluorescence was significantly enhanced and connected into a fibrous structure. The quantitative statistics showed that the fluorescence intensity of Type I collagen was 28.4 times higher than that of the blank control group (Figure 11C). In the early stage, Type III collagen rapidly formed a fibrous network, laying the foundation for the subsequent more stable deposition of Type I collagen and tissue remodeling. In the later stage, with the stimulation of PLLA microspheres, a large amount of newly generated Type III collagen was produced to fill facial depressions.

In the fluorescence staining image of Sculptra^®^ filler, in the early stage, perhaps due to the overly large encapsulation formed by the material, the growth and distribution of collagen were very uneven. In the later stage, it gradually became evenly distributed. Green fluorescence appeared around the border of the PLLA material, and Type III collagen was also dominant, with less Type I collagen generated. The fluorescence intensity of Type III collagen at 15 days (Supplementary Figure S11) was 34.4 times higher than that of the blank control group (Figure 11D). At 6 months, the fluorescence intensity of Type I collagen was 6.6 times higher than that of the blank control group (Figure 11C). Both fillers mainly stimulated the growth of Type III collagen in the first 3 months. After 3 months, there was a relatively obvious generation of Type I collagen. In comparison, the collagen growth generated by Gel@TXA-MS filler was more stable, the distribution was more even, and the generation effect was more obvious. Type I collagen and Type III collagen were 4.3 times and 1.56 times higher than those of Sculptra^®^ filler, respectively. Therefore, Gel@TXA-MS filler has a significant effect on stimulating collagen production and will play a long-term anti-aging role [67].

## Conclusion

In this study, the facial filler (Gel@TXA-MS) was formed by crosslinked HA and collagen, and loaded with porous PLLA microspheres and TXA. The Gel@TXA-MS fillers demonstrated superior immediate filling efficacy, robust collagen regeneration and low immune response during PLLA microsphere degradation. Type III collagen levels increased rapidly, reaching 34.4-fold and 1.56-fold higher than those of the control and Sculptra^®^ groups, respectively, on day 15. Six months after subcutaneous injection, the area ratio of newly formed collagen fiber bundle at the injection site reached 51.4%, with Type I collagen production being 28.4-fold higher than that of the blank control group and 4.3-fold higher than that of the Sculptra^®^ group. The honeycomb-like structure of the PLLA microspheres promoted ‘penetrating collagen growth’, facilitating targeted correction of facial depressions and wrinkles. Gel@TXA-MS demonstrates strong potential as an anti-aging dermal filler for esthetic applications with high safety, excellent volumizing performance and a long-lasting effect of 2–3 years.

## Supplementary data

Supplementary data are available at *Regenerative Biomaterials* online.

## Funding

None declared.

*Conflicts of interest statement.* There are no conflicts to declare.

## Supplementary Material

rbaf049_Supplementary_Data
