# Supplementary material for: Porous PLLA microspheres dispersed in HA/collagen hydrogel as injectable facial fillers to enhance aesthetic effects
Source: Regen Biomater. 2025 May 23;12:rbaf049. doi: 10.1093/rb/rbaf049 (PMC12187068; doi:10.1093/rb/rbaf049)
Supplement: rbaf049_Supplementary_Data [file rbaf049_supplementary_data.docx]

Porous PLLA Microspheres Dispersed in HA/Collagen Hydrogel as Injectable Facial Fillers to Enhance Aesthetic Effects

Miaoran Zhao^1^, Shuhua Chang^1^*, Yunpeng Wang^1^, Jun Cao^1^, Yuji Pu^1^, Bin He^1^*, Shengsheng Pan^2^*

^1^ National Engineering Research Center for Biomaterials, Collage of Biomedical Engineering, Sichuan University, Chengdu 610065, China

^2^ Department of Plastic Surgery, First Affiliated Hospital of Wenzhou Medical University, Wenzhou 325000, China

The Injectability of the Gel@TXA-MS Filler


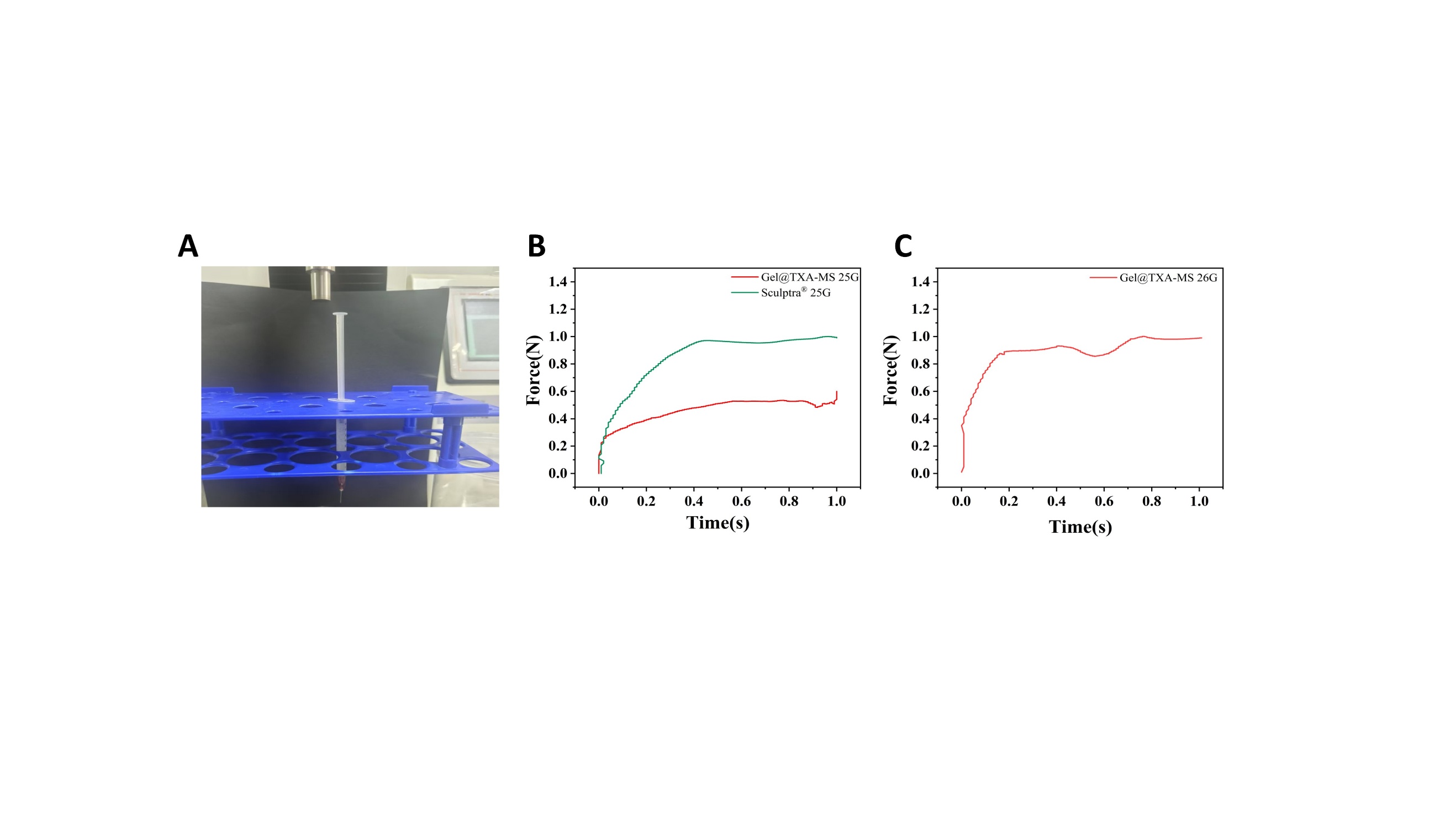


Figure S1: The universal testing machine to measure the extrusion thrust of the filler.

The Standard Curve of TXA


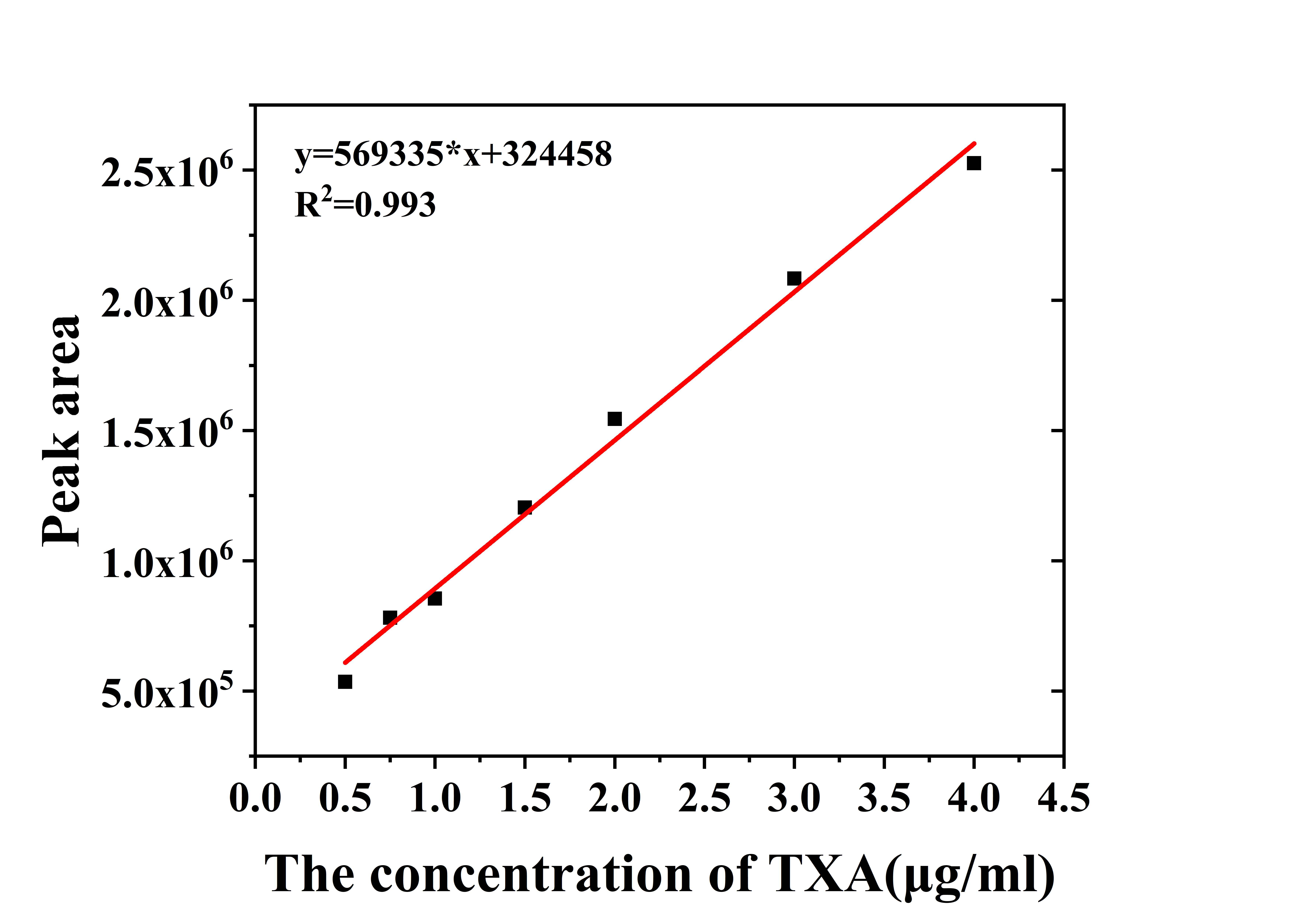


Figure S2. Standard curve of TXA determined by HPLC.

Injection Diagram of the Skin on the Back of the Experimental Rat


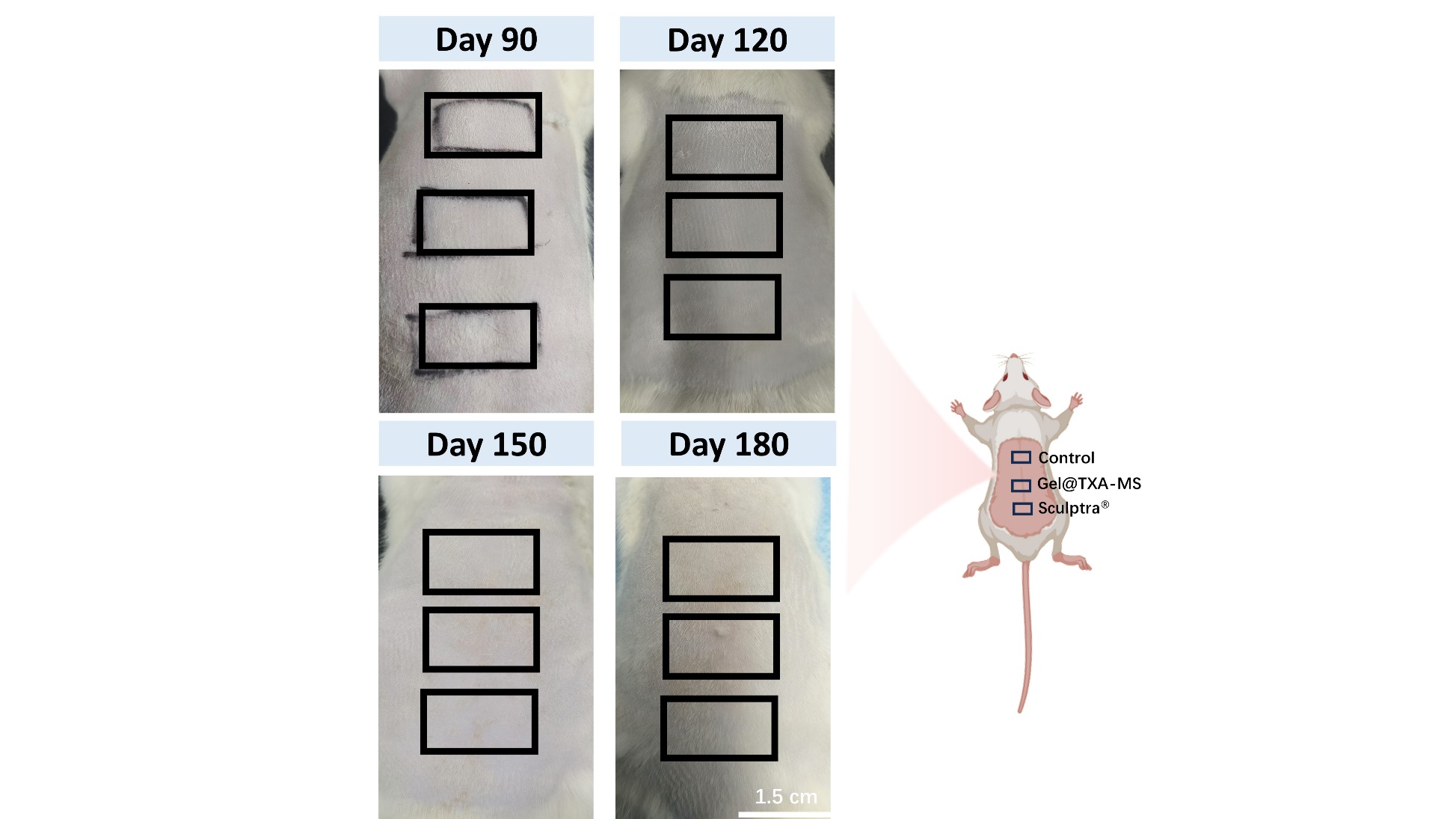


Figure S3. The diagram of immediate filling effect in animal experiments.

H&E Staining and Masson Staining


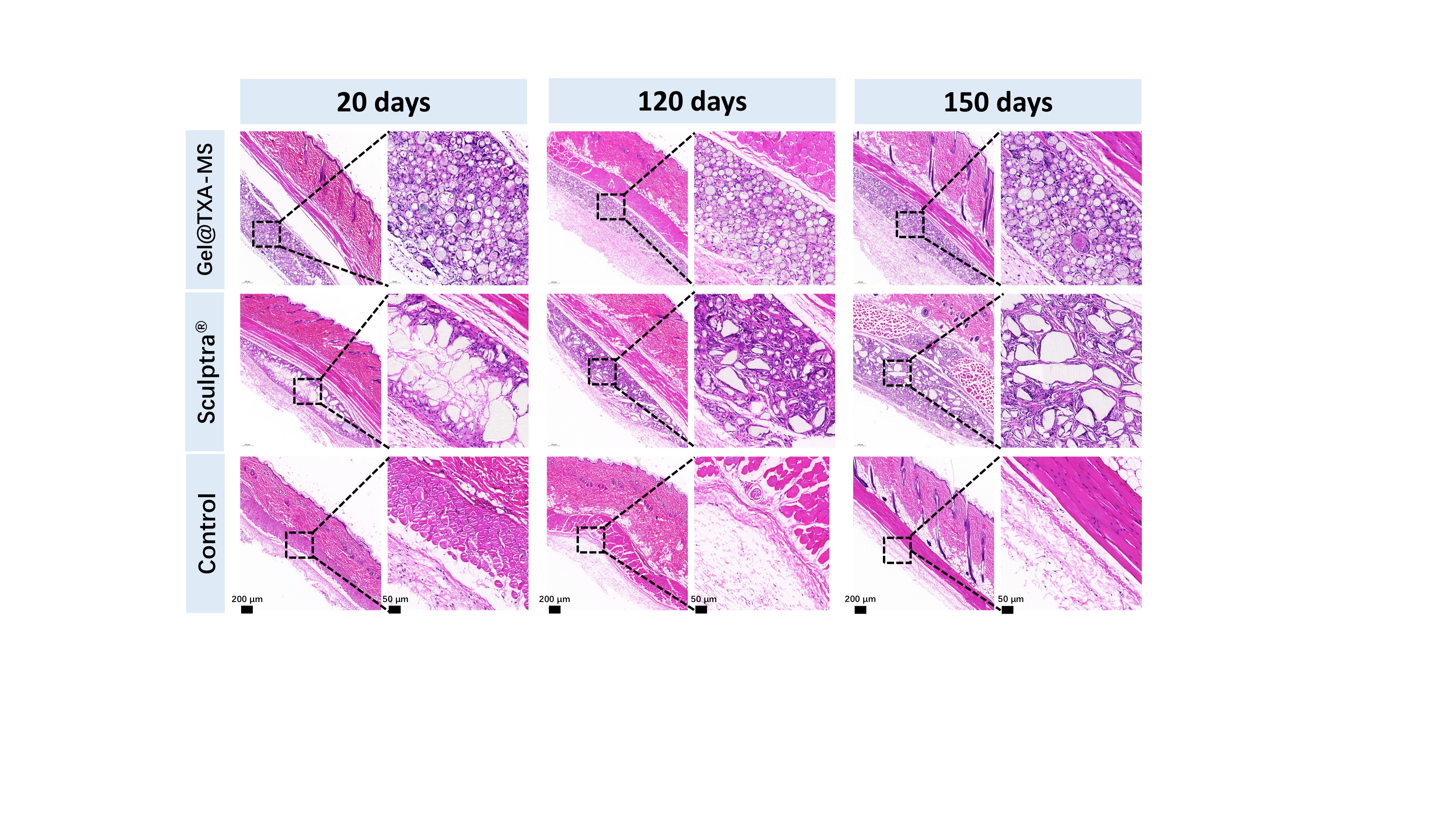


Figure S4. Scanned images of H&E staining on the 20th, 120th and 150th days.


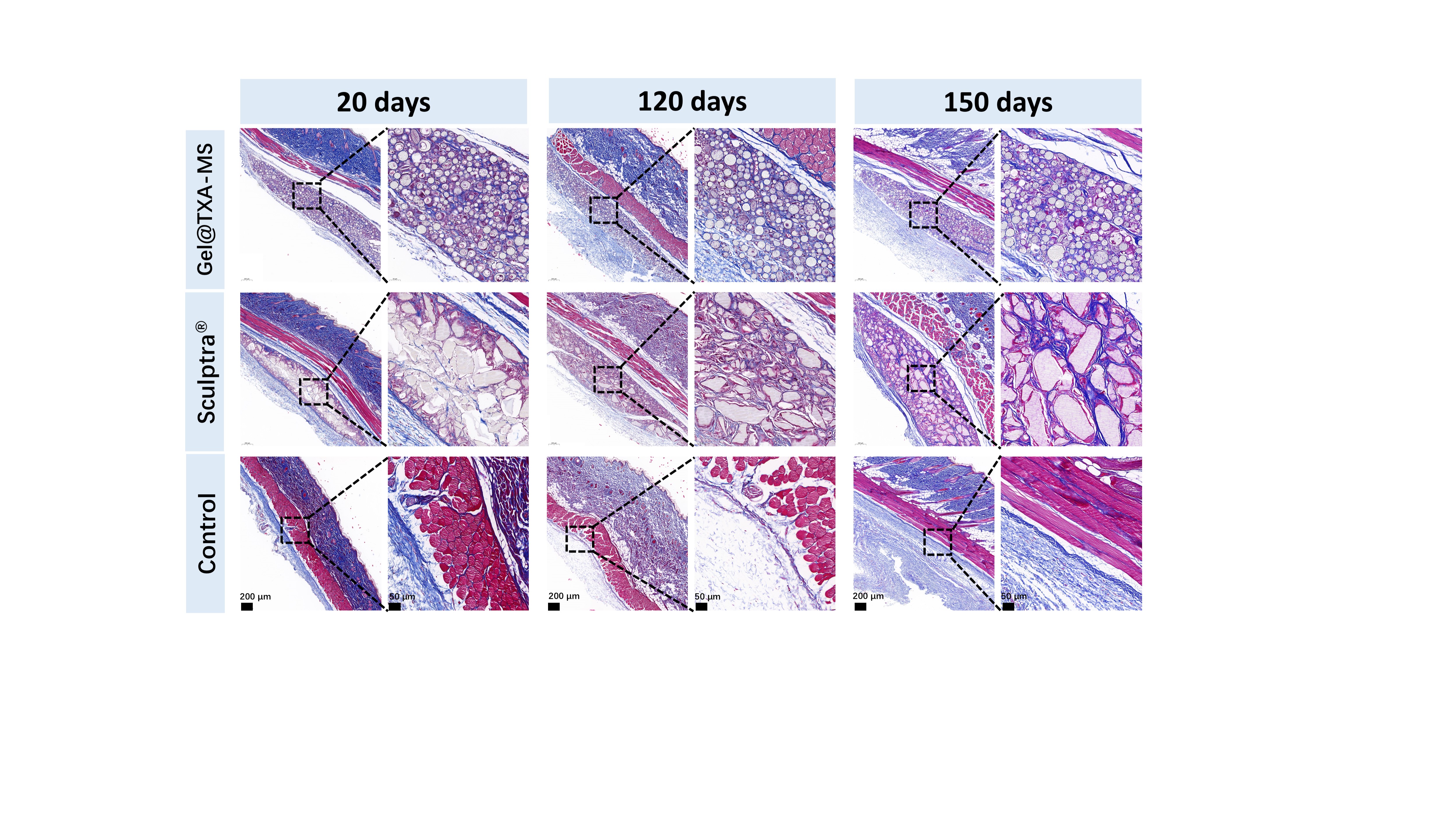


Figure S5. Scanned images of Masson staining on the 20th, 120th and 150th days.

Cytotoxicity


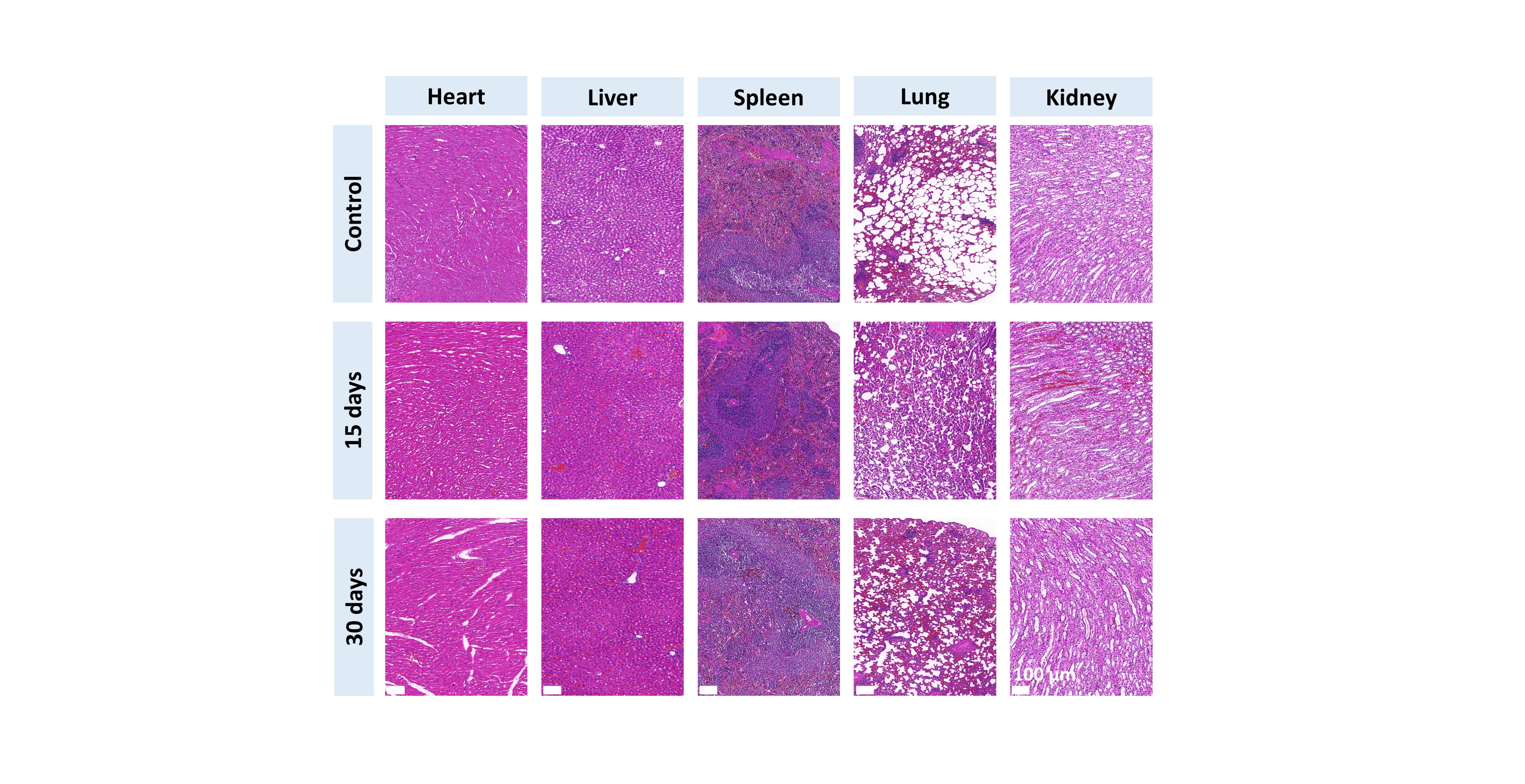


Figure S6. H&E staining images of the main organs (heart, liver, spleen, lung, and kidney) of the experimental mice at 15 and 30 days


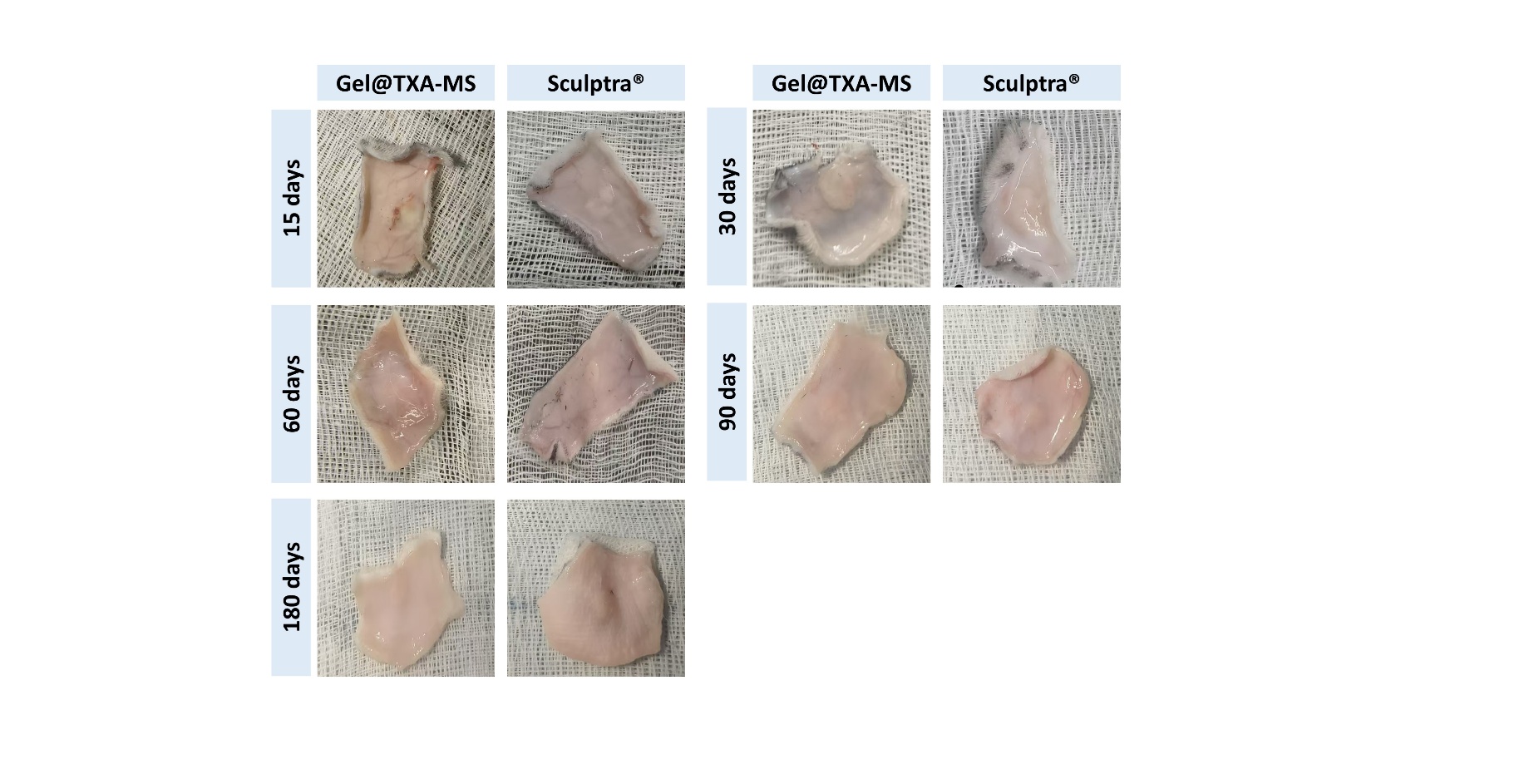


Figure S7. Internal morphology images of the full-thickness skin at the site where the material was injected on the back of the rats at 15, 30, 60, 90 and 180 days.

Fluorescence Staining Images of CD68、IL-6、Ⅰ collagen、Ⅲ collagen


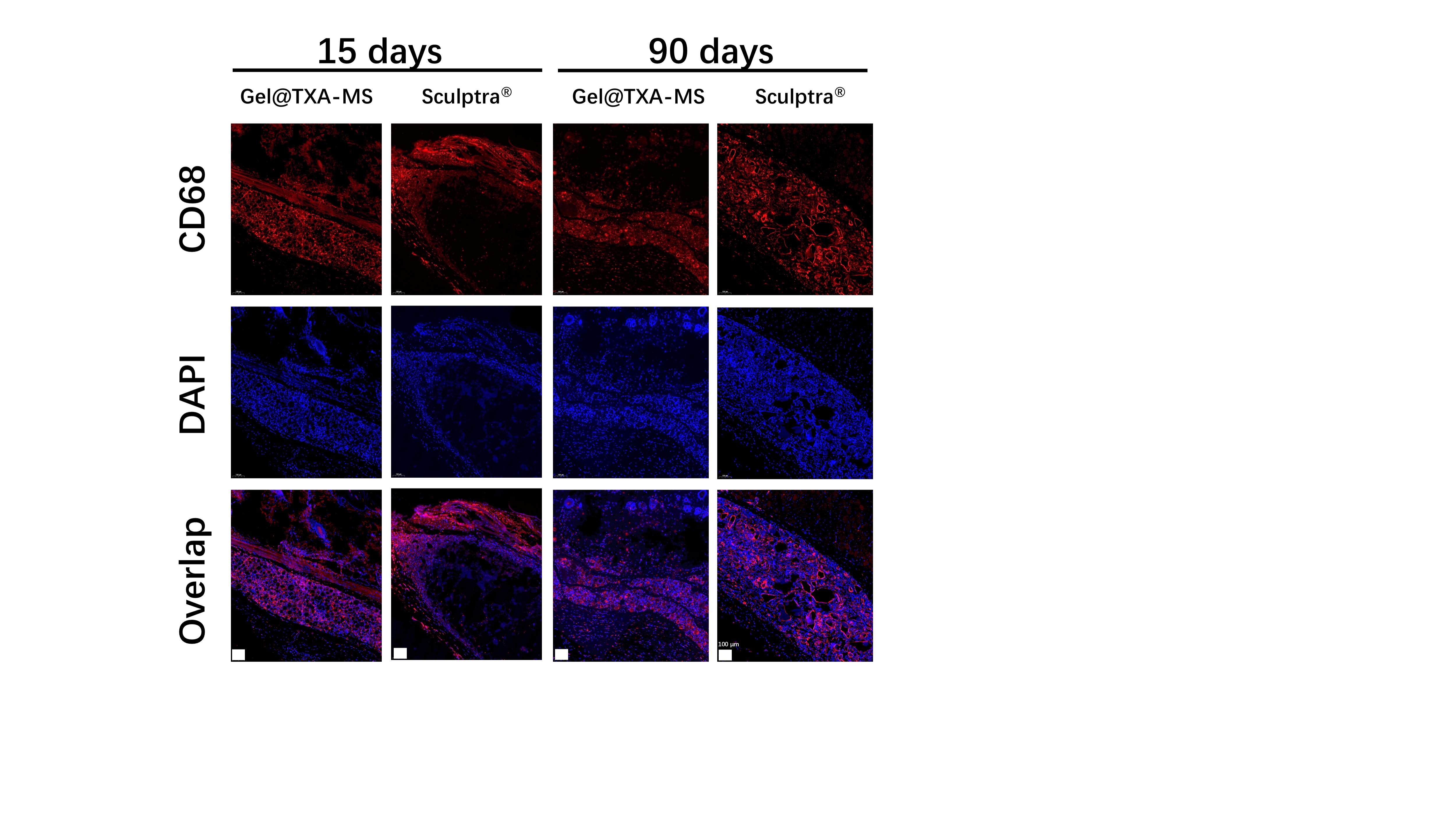


Figure S8. Fluorescence staining images of CD68 on the 15th and 90th days.





Figure S9. Fluorescent staining image and quantitative analysis image of IL-6.


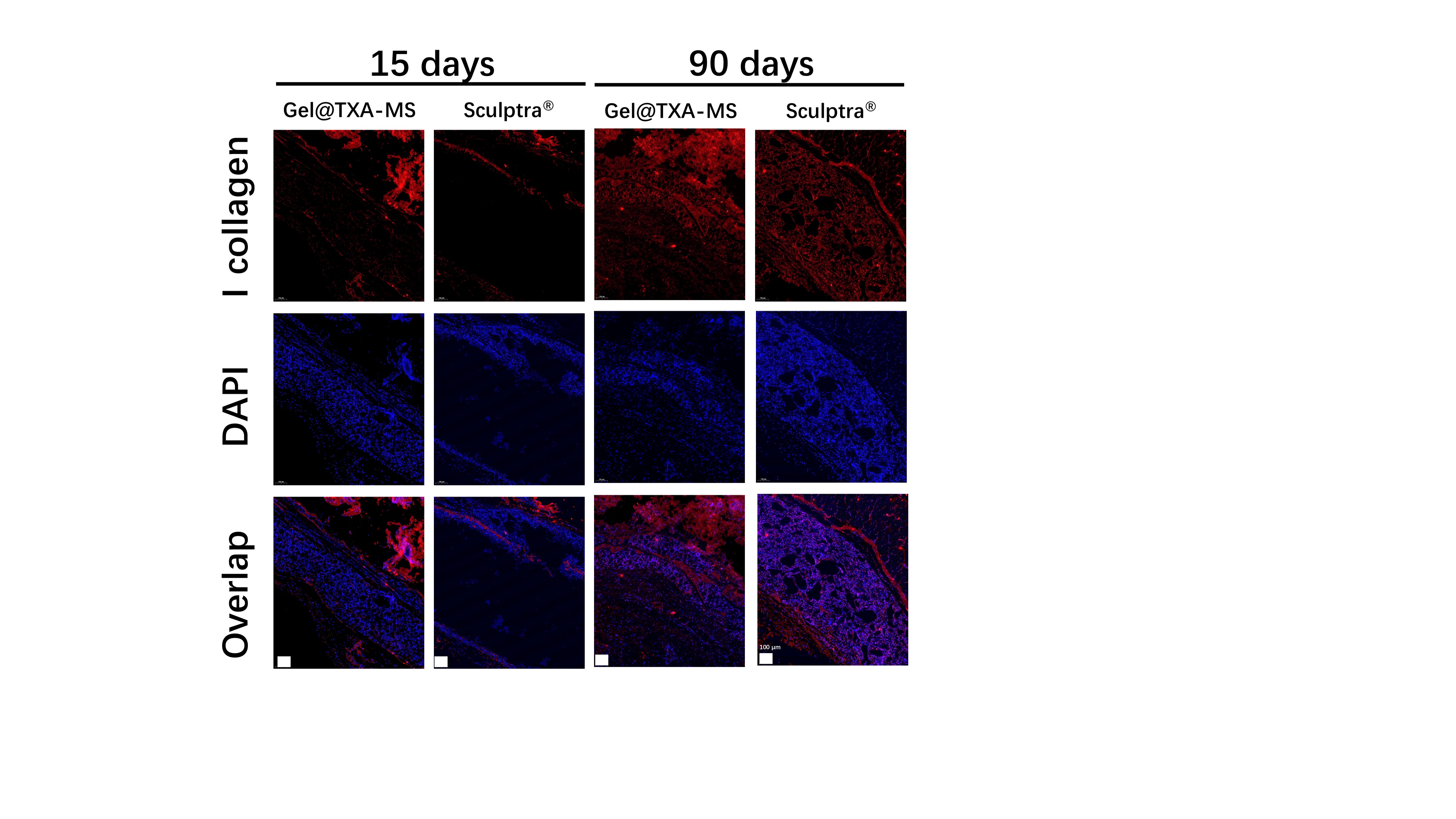


Figure S10. Fluorescence staining images of Type Ⅰ collagen on the 15th and 90th days.


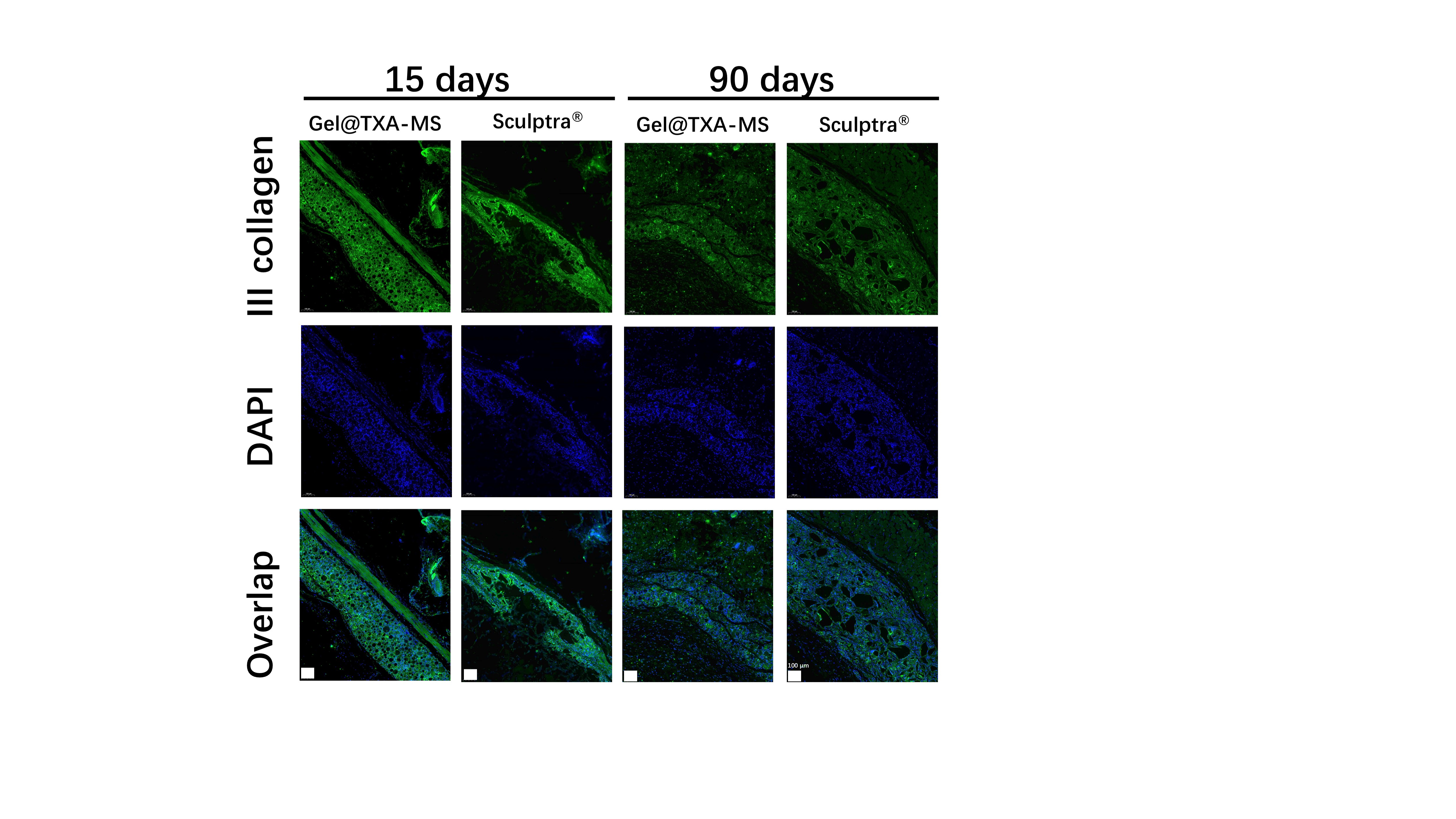


Figure S11. Fluorescence staining images of Type Ⅲ collagen on the 15th and 90th days.
